# Integrative models explain the relationships between species richness and productivity in plant communities

**DOI:** 10.1038/s41598-019-50016-3

**Published:** 2019-09-24

**Authors:** Zhenhong Wang, Alessandro Chiarucci, Juan F. Arratia

**Affiliations:** 1 Key Laboratory of Subsurface Hydrology and Ecological Effects in Arid Regions, Ministry of Education, Chang’an University, Xi’an, China; 2 School of Environmental Science and Engineering, Chang’an University, Xi’an, 710064 China; 3 Department of Biological, Geological and Environmental Science, University of Bologna, Via Irnerio, 42-40126 Bologna, Italy; 4 AGMUS Institute of Mathematics, Caribbean Computing Center for Excellence, 21150 San Juan, Puerto Rico USA

**Keywords:** Community ecology, Biodiversity, Ecological modelling

## Abstract

The relationship between plant productivity and species richness is one of the most debated and important issues in ecology. Ecologists have found numerous forms of this relationship and its underlying processes. However, theories and proposed drivers have been insufficient to completely explain the observed variation in the forms of this relationship. Here, we developed and validated integration models capable of combining twenty positive or negative processes affecting the relationship. The integration models generated the classic humped, asymptotic, positive, negative and irregular forms and other intermediate forms of the relationship between plant richness and productivity. These forms were linked to one another and varied according to which was considered the dependent variable. The total strengths of the different positive and negative processes are the determinants of the forms of the relationship. Positive processes, such as resource availability and species pool effects, can offset the negative effects of disturbance and competition and change the relationship. This combination method clarifies the reasons for the diverse forms of the relationship and deepens our understanding of the interactions among processes.

## Introduction

Plant productivity and species richness are two fundamental properties of plant community structure and functioning. The relationships between plant productivity and species richness at different spatial scales have been the subject of numerous studies and continue to be a controversial ecological topic^1–5^. Much of the controversy is historically focused on interpretations of the relationship between species richness and community productivity as well as in the interpretation of the underlying mechanisms. Basically, there are two potential relationships: (1) the plant productivity-species richness relationship (PSRR), in which plant productivity is considered to be an independent variable and species richness as a dependent variable, which elucidates how patterns of plant diversity are affected by plant productivity, and (2) the species richness-plant productivity relationship (SRPR), i.e., the feedback relationship to PSRR, in which species richness is conversely considered to be an independent variable and plant productivity as a dependent variable, which clarifies the effects of plant diversity on plant productivity related to ecosystem functioning, stability, and services^4–6^.

The most typical form of the PSRR is a humped (or unimodal) curve, with richness first increasing and then decreasing with increasing productivity^3,7,8^. The most typical form of the SRPR is an asymptotic or positive form, i.e., plant productivity is positively affected by species richness until a certain value of the latter is reached, above which productivity maintains a constant value^9,10^. The mechanisms proposed to explain the PSRR and SRPR include many processes, theories, and hypotheses, such as the species-energy theory^4,11,12^; the metabolic rate of ecosystems^13,14^; the interspecific competitive exclusion hypothesis^15–18^; the assemblage-level thinning hypothesis^19–21^; the resource ratio theory^4^; the species pool effect^22^; dispersal limitation^23,24^; disturbance^25,26^; resource availability^4,27^; environmental heterogeneity^28,29^; and the selection effect, the complementary effect, density effects, and the biodiversity and ecosystem functioning theory^9^.

However, the typical forms of the PSRR and SRPR have often been challenged because results from assembly experiments, field investigations, and mathematical modelling have indicated other forms, including negative monotonic, irregular, unpredictable, unrelated, level, or even U-shaped patterns^5,8^. Over the past several decades, ecologists have been intensely debating the most general pattern among these forms and underlying mechanisms^7,19,21,22,30–34^. Although ecologists have adopted standardized and consistent approaches to reconcile the differences in the forms and to clarify the underlying causes, various forms of the PSRR and SRPR still arise, leading to debates^8,35–39^. Some authors suggest that ecologists should focus on conducting integrative analyses of the causes of the mechanisms controlling the PSRR and SRPR since they are variable and complex, being governed by many abiotic and biotic factors and affected by scale dependence^39,40^. Ecological processes affecting plant productivity, species richness, and their relationship often have either a positive or negative effect, and some even have the two effects simultaneously^4,7,27^. Therefore, completely understanding the PSRR and SRPR and their underlying mechanisms necessitates (1) the clarification of the respective effects of the different processes; (2) the use of suitable theoretical methods to combine the respective effects of these processes into a logical comprehensive effect on the PSRR and SRPR; and (3) the use of observed data to verify the derived forms by using integration models and to identify the underlying mechanisms.

Some previous studies have been concerned with the use of integrative models to predict the PSRR and SRPR forms, such as the non-equilibrium interaction model^41^, multispecies patch-occupancy models^42^, the resource-ratio model^16^, the habitat template model^43^, and the modified neutral model^44^. However, the methods used in these studies do not adhere to the three criteria discussed at the end of the preceding paragraph. Specifically, these studies were often based on elegant models with few variables (in contrast to the large number of variables in the real world) to derive the dominant forms of the PSRR and SRPR. Once the dominant form is challenged, theoretical studies need new models to explain other forms of the PSRR and SRPR. Therefore, in the field, there are many models that cannot completely explain all forms of the PSRR and SRPR. For example, a mechanistic model that identifies plants that are able to use limited soil nutrients with increasing diversity in ecosystems generates a positive form of the SRPR^45^, whereas a modified multispecies patch-occupancy model reveals negative, positive, and humped forms of the PSRR along disturbance and productivity gradients^46^. Recently, Grace *et al*.^39^ structured a causal network of the humped form of the PSRR, in which the humped form was determined to be a dominant form affected by multiple processes, and the causes and patterns of the PSRR were evaluated using structural equation modelling. Dramatically, this formal analysis rejected the humped form of the PSRR and revealed the effects of additional active processes. Trait-based models described by Bayes’ Theorem were also used to explain ecosystem restoration with some derived forms of the PSRR and SRPR^47^.

In this study, we did not a priori assume any documented forms of the PSRR or SRPR from previous studies as dominant forms to be derived by models. Conversely, we combined all crucial positive and/or negative processes widely found by ecologists in their studies to affect the PSRR and SRPR into a set of integration models based on theories of differential equations and ecological dynamical systems. We changed the values of the model parameters to regulate the strengths of these crucial processes to derive the forms of the PSRR and SRPR and identify underlying mechanisms. To prevent the causes from being confused with consequences, we explicitly defined plant species richness (*s*) as a dependent variable and plant productivity (*P*) as an independent variable to establish the PSRR models for the quantification of the effects of plant productivity on species richness. Next, by using the PSRR models, we derived models of the SRPR, i.e., the feedback relationships to the PSRR, in which plant productivity (*P*) was conversely considered as a dependent variable and species richness as an independent variable. We suggested that (1) ecological processes that have a positive or negative effect on the PSRR and SRPR vary temporally or spatially; (2) the processes that have a strongly positive effect at one productivity or richness level might have a weakly positive or negative effect at a different productivity or richness level; and (3) the combination of all positive and/or negative process effects radically determines the forms of the PSRR and SRPR. First, we assessed the positive and/or negative effects of crucial processes that have been widely considered in the literature to regulate plant productivity, species richness, and the relationships between them. Second, we noted how the models might combine the effects of these crucial processes and other processes or factors. Third, we used the integration models to derive the forms of the PSRR and SRPR at local and regional scales and verified the forms at the local scale using observed data.

## Results

### The PSRR and SRPR at the local scale

#### The PSRR

**Figure 1:**
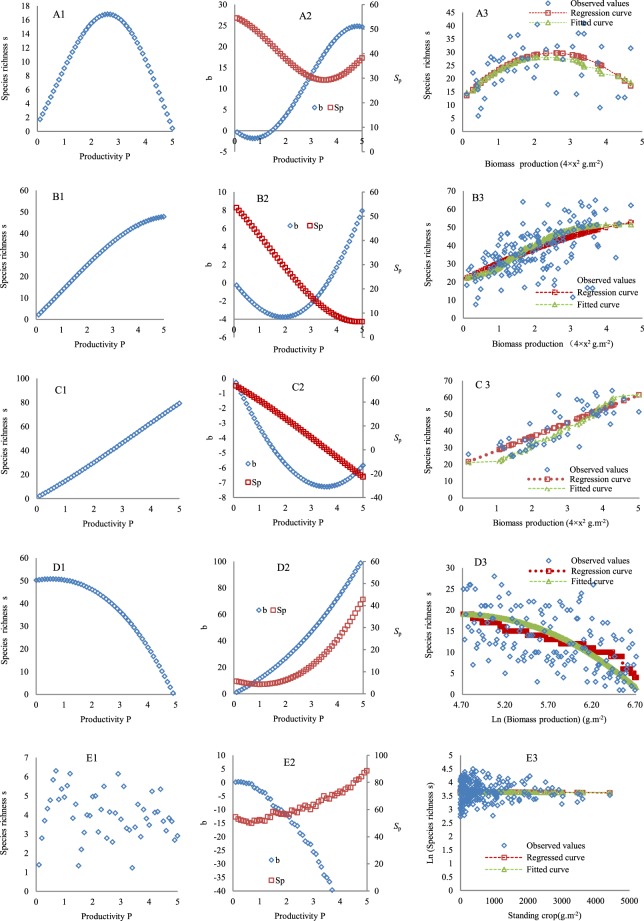
Typical forms of the PSRR. (**A1**–**E1**) represent the humped, asymptotic, positive, negative, and irregular forms, respectively. (**A2**–**E2)** show the dynamics of IICE (*b*) and the potential species-pool effect (*S*_*p*_) for the five forms. (**A3**–**E3**) show the observed species richness along a productivity gradient at local sites in Germany, the Czech Republic, Russia^38^, the USA^57^, and Australia^58^, respectively. Regression curves were fitted based on the observed species richness. Fitted curves were drawn using the predicted species richness (obtained using the second value in each cell in the data columns marked with # in Supplemental Table 1 being substituted into Eq. 10).

When the first value in each cell in the data columns marked with # in Supplemental Table 1 was substituted into Eqs 10 and 11, the five typical forms of the PSRR with the dynamics of the potential species-pool effect (*S*_*p*_) and intra-and interspecific competition effects (IICE, *b*) were derived (Fig. 1). The first is a humped form (Fig. 1A1), with species richness (*s*) first increasing along with increasing plant productivity (*P*) and then decreasing. The dynamics of *S*_*p*_ show the sunken form (Fig. 1A2). The reason for the sunken *S*_*p*_ is that when *s* gradually increases, the actual contribution of the species pool to the target community is increased, but the size (*aA*) of the species pool is invariable; therefore, *S*_*p*_ becomes small (Eq. 2). Conversely, when *s* declines at the right side of the humped form (Fig. 1A1), the corresponding *S*_p_ increases (Fig. 1A2). However, *b* presents a form similar to a capital S in association with *P* and *s*. Overall, the combined positive processes regulating the PSRR are dominant at the rising section of the humped PSRR, but combined negative processes act after the intermediate *P* level, forming the humped pattern. The second is an asymptotic form (Fig. 1B1), with *s* continually increasing along with *P* and reaching its maximum value at the highest *P* level. *S*_*p*_ gradually declines, and *b* increases with increasing *P* (Fig. 1B2). The process parameters *m*_1_ and *m*_2_ are greater for the asymptotic form than for the humped form (Supplemental Table 1). The relatively higher strength of the combined positive processes than the combined negative ones generates the asymptotic form. Third is a positive form (Fig. 1C1), which is similar to the asymptotic form, but the end of the curve is different. The corresponding *S*_*p*_ and *b* are also similar (Fig. 1C2). However, the process parameters *m*_1_ and *m*_2_ are greater for the positive form than for the asymptotic form (Supplemental Table 1). For this form, combined positive processes are always dominant. Fourth is a negative form (Fig. 1D1), in which *s* continually decreases. Both *S*_*p*_ and *b* continually increase (Fig. 1D2). Combined negative processes primarily shape this pattern. Fifth is an irregular form (Fig. 1E1). This form is irregular or unrelated to *P* because there are different disturbances to the PSRR along the *P* gradient (Supplemental Table 1). *S*_p_ shows a moderate increase along the *P* gradient, whereas *b* continuously slumps (Fig. 1E2). In Supplemental Table 1, only the parameters from which the five typical forms of the PSRR were derived are given. If the values of these parameters differ from those in Supplemental Table 1, other intermediate forms will arise (Supplemental Material 1).

Verification of the PSRR forms indicated that there were no significant differences between the observed and fitted *s* according to the *t*-tests and goodness-of-fit tests for the humped form (t = 0.34, n = 43, p = 0.72; X^2^ = 5.02, n = 43, p > 0.995; Fig. 1A3), the asymptotic form (t = 1.56, n = 153, p = 0.12; X^2^ = 26.96, n = 154, p > 0.995; Fig. 1B3), the positive form (t = 0.26, n = 45, p = 0.20; X^2^ = 18.83, n = 45, p > 0.995; Fig. 1C3), the negative form (t = 0.33, n = 147, p = 0.74; X^2^ = 82.35, n = 147, p > 0.995; Fig. 1D3), and the irregular form (t = 0.06, n = 235, p = 0.85; X^2^ = 8.24, n = 235, p > 0.995; Fig. 1E3). Thus, the five derived forms of the PSRR can fit the observed forms well. The estimated parameter values, i.e., the second value in each cell in the data columns with # in Supplemental Table 1, can to some degree represent the process strengths affecting these five observed PSRR forms.

#### SRPR

**Figure 2:**
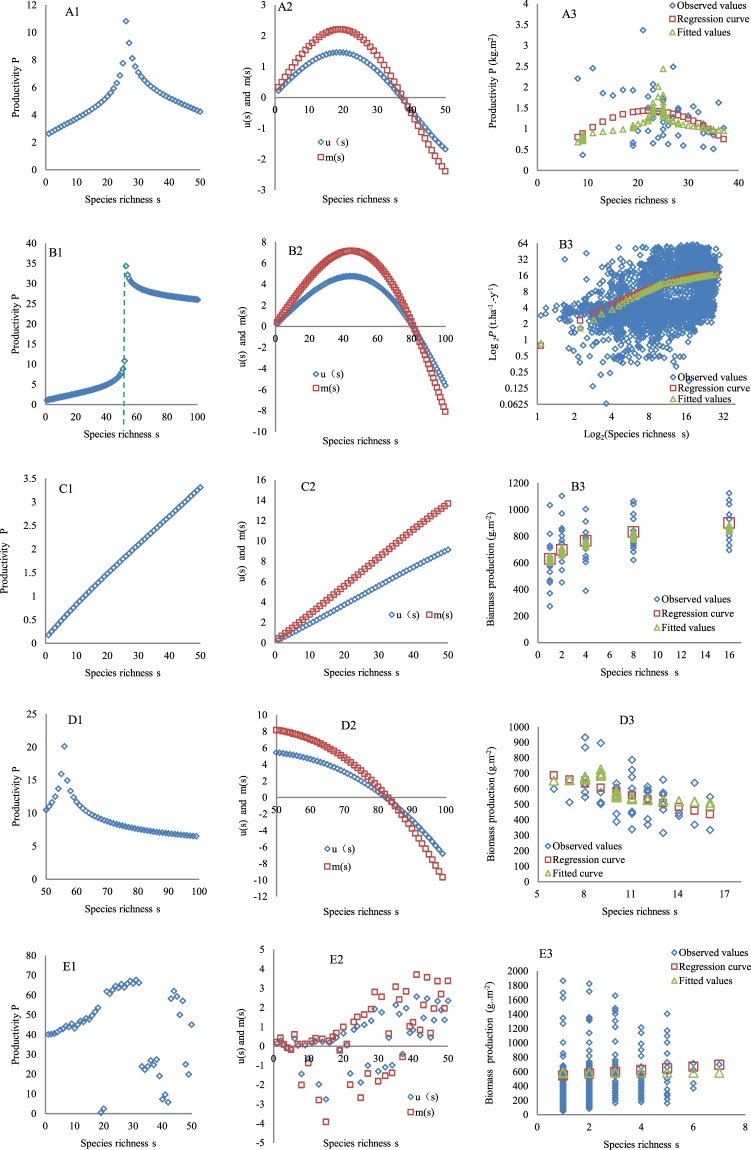
Forms of the SRPR. (**A1**–**E1**) represent the humped, asymptotic, positive, negative, and irregular forms, respectively. (**A2**–**E2**) show the dynamics of the SC effects (*u*(s)) and density effect (*m*(s)) for the five forms. (**A3**–**E3**) show the observed productivity along a species richness gradient in the grasslands of Texas^67^, forest plots around the world^56^, the floodplain of the Saale River in Germany^10^, and the plant community in Gloucestershire of the UK^59,68^, respectively. The regression curve was fitted based on the observed productivity. Fitted curves were drawn using the predicted productivity (obtained using the third value in each cell in the data columns marked with # in Supplemental Table 1 being substituted into Eq. 21).

(1) SRPR with monotonically increasing *s*. The forms of the SRPR were also derived (Fig. 2A1–E1) after a set of the same parameter values as those used to derive the PSRR in Fig. 1 (the first value in each cell in the data columns marked with # in Supplemental Table 1) had been substituted into Eq. 21. The SRPR also shows five forms, i.e., the humped, asymptotic, positive, negative and irregular forms also observed for the PSRR, but they do not look as typical as the shapes of the curves for the PSRR. The SRPR, i.e., the feedback to the humped PSRR in Fig. 1A1, is still humped (Fig. 2A1). However, its peak tapers more in comparison to that in Fig. 1A1. Both sampling and the complementarity (SC) effects (*u*(s)) and density effects (*m*(s)) affecting the SRPR indicate a rounded humped form (Fig. 2A2) and become negative at the end of the humped form, suggesting that the effects of *u*(s) and *m*(s) disappear at high *s* levels. The SRPR associated with the asymptotic PSRR in Fig. 1B1 can be considered to have two forms (Fig. 2B1): a positive form with *s* on the x-axis ranging from 0 to 50 units (in front of the dotted line) and a similarly asymptotic form with *s* ranging from 0 to 80 units. Although *u*(s) and *m*(s) are also the humped forms, their peak values occur at a higher *s* level (Fig. 2B2). The SRPR related to the positive PSRR in Fig. 1C1 is still positive (Fig. 2C1), and *u*(s) and *m*(s) are also positive (Fig. 2C2), indicating that the integrated positive processes are dominant. For the SRPR related to the negative PSRR in Fig. 1D1, after a small peak in *P*, *P* shows a gradual decrease with increasing *s* beginning from a high primary *s* (Fig. 2D1). Correspondingly, both *u*(s) and *m*(s) simultaneously decline (Fig. 2D2), and the high primary *s* causing strong IICE is key for the *u*(s) and *m*(s) and corresponding SRPR form. The SRPR related to the irregular PSRR in Fig. 1E1 is also irregular because of the different intensities of disturbance (Fig. 2E1). The corresponding *u*(s) and *m*(s) in Fig. 2E2 are not as regular as those in Fig. 2A2–D2 due to disturbance. Nevertheless, there is still a weak increasing trend for *u*(s) and *m*(s), with their positive effects on *P* being offset by disturbance (Fig. 2E2). Overall, these changes in the SRPR forms are identical to those for *u*(s) and *m*(s).

Verification of the SRPR with monotonically increasing *s* indicated that no significant differences occurred between the observed and fitted *P* for the asymptotic form (t = 0.23, n = 50, p = 0.82; X^2^ = 1.97, n = 50, p > 0.995; Fig. 2B3)and the positive form (t = 0.97, n = 57, p = 0.33; X^2^ = 48.54, n = 57, p > 0.10; Fig. 2C3) based on both the t-test and goodness-of-fit test. The t-tests also showed no significant difference between the observed and fitted P for the humped form (t = 0.87, n = 54, p = 0.38; Fig. 2A3), the negative form (t = 0.53, n = 44, p = 0.62; Fig. 2D3), and the irregular form (t = 1.47, n = 164, p = 0.14; Fig. 2E3). However, a significant difference existed between the observed and fitted P for the humped form (X^2^ = 1817.08, p < 0.005; Fig. 2A3), the negative form (X^2^ = 155.06, p < 0.05; Fig. 2D3), and the irregular form (X^2^ = 345.24, p < 0.01; Fig. 2E3) based on the goodness-of-fit test. Although the correspondence of the derived SRPR to the observed SRPR was not as good as that of the derived PSRR to the observed PSRR, the derived SRPR and the estimated parameter values, i.e., the third value in each cell in the data columns with # in Supplemental Table 1, can to some degree explain the observed forms and the process strengths affecting these observed forms. The diverse fitted curves in Fig. 2A3–E3 also indicate the high plasticity of the models of the SRPR.

**Figure 3:**
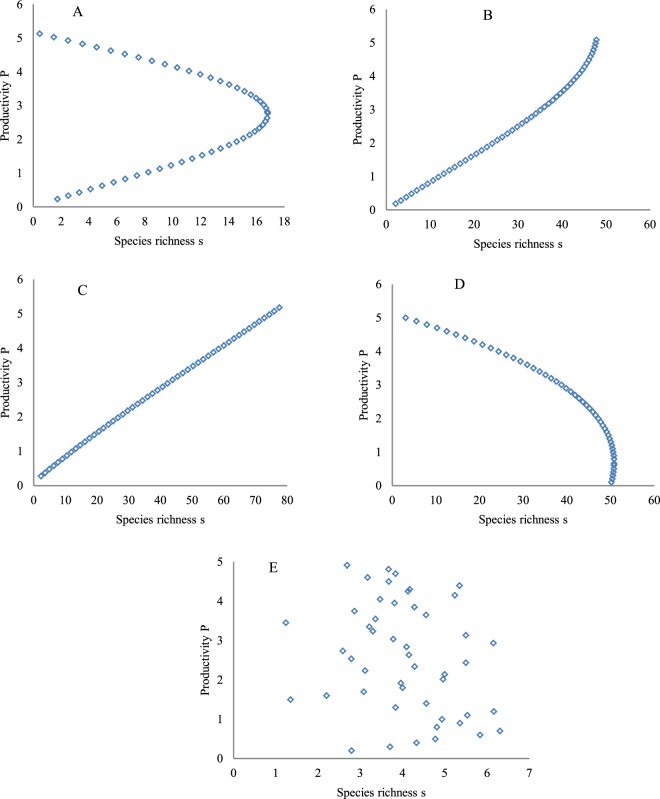
The SRPR with *s* continually presenting non-monotonic increases. These figures were created based on the calculation results of Eq. 10, i.e., species richness as inputs to Eq. 21 to calculate plant productivity for a transformation of dependent variables and independent variables. (**A1**–**E1**) show the humped, positive, asymptotic, negative and irregular forms, respectively, generated by substituting the derived species richness in Fig. 1A1–E1 into Eq. 21.

(2) SRPR with non-monotonically increasing *s*. Five SRPRs with non-monotonically increasing *s* were derived (Fig. 3A–E) when the species richness in Fig. 1A1–E1 was substituted into Eq. 21. In other words, the calculation results of Eq. 10, i.e., species richness, were used as inputs in Eq. 21 to calculate plant productivity. This resulted in the transformation of dependent variables and independent variables, or feedback to the PSRR. In Fig. 3A,B,D,C,E, species richness (*s*) on the x-axis continually shows a non-monotonic increase. Specifically, in Fig. 3A, species richness decreases when species richness increases to 16 on the x-axis, but plant productivity on the y-axis continually increases with increasing and then decreasing species richness. Species richness begins to stabilize when it approaches 50 units in Fig. 3B, but, correspondingly, plant productivity on the y-axis always increases. Species richness on the x-axis always increases from 1–80 species in Fig. 3C, and a decrease in species richness is not noted. Correspondingly, plant productivity shows a continual increase. Species richness always increases from 1 to 50 species on the x-axis in Fig. 3D, and, conversely, plant productivity continually decreases with increasing species richness. With increasing species richness on the x-axis in Fig. 3E, plant productivity along the y-axis fluctuates due to different intensities of disturbance. In fact, species richness along the x-axis also fluctuates in Fig. 3E, as shown in Fig. 1E1, but the fluctuation in species richness is hidden by mapping. A comparison of Fig. 3A–E with Figs 1A1–E1 indicates that Fig. 3A–E are simply products of coordinate rotation of Fig. 1A1–E1 at 90°. The dynamics of IICE and *S*_p_ corresponding to Fig. 3A–E are the same as those for Fig. 1A2–E2 even if the coordinates are rotated.

### The PSRR and SRPR at the regional scale

**Figure 4:**
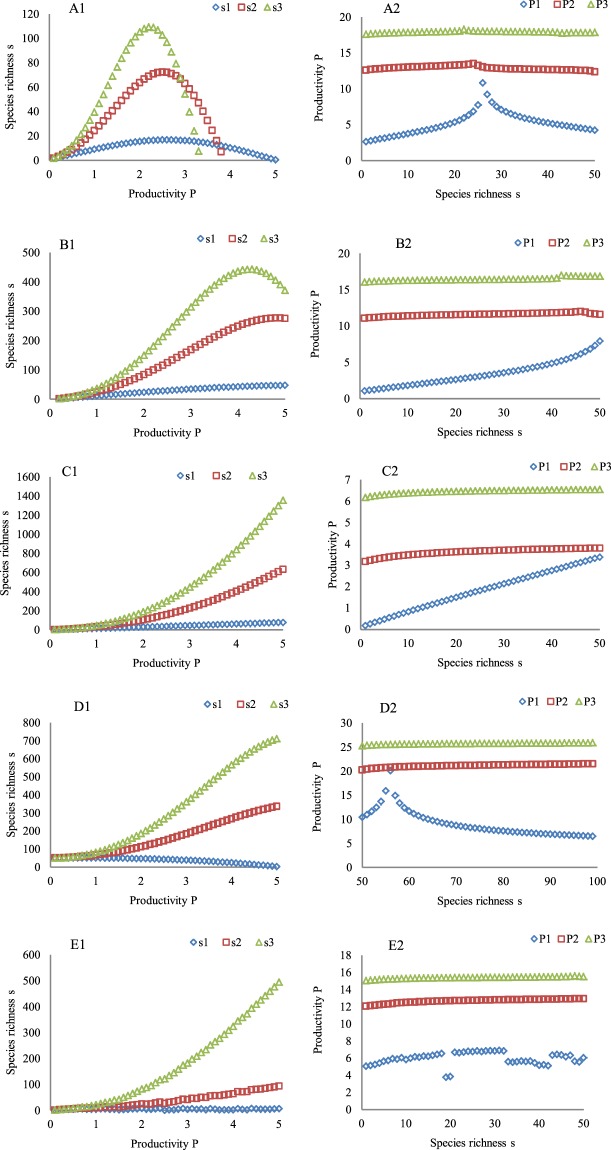
Changes in the forms of the PSRR and SRPR with the intrinsic rate of species richness (*m*_1_), resource availability (*m*_2_), and effect coefficient (*a*) related to the species pool and environmental heterogeneity (*E*_h_) across different zonal forests. The addition of increments to *m*_1_, *m*_2_, *a*, and *E*_h_ leads to the formation of gradients in *m*_1_, *m*_2_, *a*, and *E*_h_, respectively, starting from DCF across ENF, TDBF, ECBF, EBF, and TRF from the north to south in Russia, Japan and the Philippines^48^. These gradients are created by assigning increments of 0.05, 0.05, 0.02, and 0.06 to *m*_1_, *m*_2_, *a*, and *E*_h_, respectively, spanning over one productivity unit on the x-axis for the *s*_2_ curve from (**A1**–**E1**) and 0.1, 0.1, 0.03, and 1 for the *s*_3_ curve. Other parameters that are substituted into Eqs 10, 11, and 21 are equal to the first value in each cell in the data columns marked with # in Supplemental Table 1. *P*_0_ and *S*_0_ in a southern forest are assumed to be greater than those in a northern forest. The *s*_1_ curve is a control, and the values of the parameters used to derive it are the same as those used to derive the five PSRR and SPRP forms in Figs 1A1–E1 and 2A1–E1. (**A1**) the humped shape in DCF changes into a humped shape with a high *s* peak (s_2_ and s_3_). (**B1**) the asymptotic shape is maintained with a high s peak (*s*_2_) and changes into a shape that is close to humped (s_3_). (**C1**) a positive shape is maintained (s_2_ and s_3_). (**D1**) the negative shape changes into positive shapes (s_2_ and s_3_). (**E**) the irregular shape changes into positive shapes (s_2_ and s_3_). (**A2-E2**) show the SRPR forms, i.e., the feedbacks to (**A1**–**E1)**, which change from non-typical humped, positive, negative, and irregular forms to level forms. s1, s2, and s3 represent different curves of species richness, and P1, P2 and P3 represent different productivity curves. The P1, P2, and P3 curves represent the feedbacks to the s1, s2, and s3 curves, respectively.

The local derivation in Fig. 1 indicates that the five forms of the PSRR might arise in a plant community with different strengths of ecological processes. Thus, the five PSRR forms were used as the initial forms in deciduous coniferous forests (DCF) to determine the changes in the shapes of the PSRR with increases in the intrinsic rate of species richness (IRSR, *m*_1_), resource availability (*m*_2_), and the effect coefficient (*a*) (Supplemental Table 1) along the assumed transect across evergreen needle-leaf forests (ENF), temperate deciduous broad-leaved forests (TDBF), evergreen coniferous and broad-leaved mixed forests (ECBF), evergreen broad-leaved forests (EBF), and tropical rainforests (TRF) in Russia, Japan and the Philippines^48^. The results indicate that the combined positive processes were strengthened. All five initial forms of the PSRR in the DCF changed into other forms except for the positive form (Fig. 4C1). The highest *s* in each of the initial forms was low; however, with different increases in *m*_1_, *m*_2_, *E*_h_, and *a*, the highest *s* showed significant changes (Fig. 4A1–E1). Notably, the initial irregular form of the PSRR was observed owing to the remarkably different disturbances (Fig. 4E1). However, after different increments had been added to *m*_1_, *m*_2_, *E*_h_, and *a* across these forests, the initial irregular form became a positive form (Fig. 4E1). This suggests that positive ecological processes weaken or offset the negative effects of disturbance on *s* and the PSRR.

The SRPR forms with increments added to *m*_1_, *m*_2_, *E*_h_, and *a* were also diverse (Fig. 4A2–E2), including the humped, level, positive, negative, and irregular forms, which are not as typical as those shown in Fig. 4A1–E1. The humped form occurs with small values of *m*_1_, *m*_2_, *E*_h_ and *a*, but the humped form changes into a level line when large increments are added to the four parameters (Fig. 4A2). Similarly, the positive SRPR forms (the feedbacks to the asymptotic and positive PSRR) change into level forms when greater increments are added to *m*_1_, *m*_2_, *E*_h_, and *a* (Fig. 4B2,C2). For the SRPR forms corresponding to the negative and irregular PSRR, a transition from both the similarly negative form and irregular form to level forms is noted with increments being added to *m*_1_, *m*_2_, *E*_h_, and *a* (Fig. 4D2,E2). These results indicate that the strengthened effects of these positive processes significantly act on *P* (different levels of curves), but *P* does not obviously remain sensitive to increasing *s* (each curve corresponds to increasing *s* on the x-axis). These forms are different from those of the PSRR, in which changes in *s* with increasing *P* on the x-axis are very significant (Fig. 4A1–E1).

**Figure 5:**
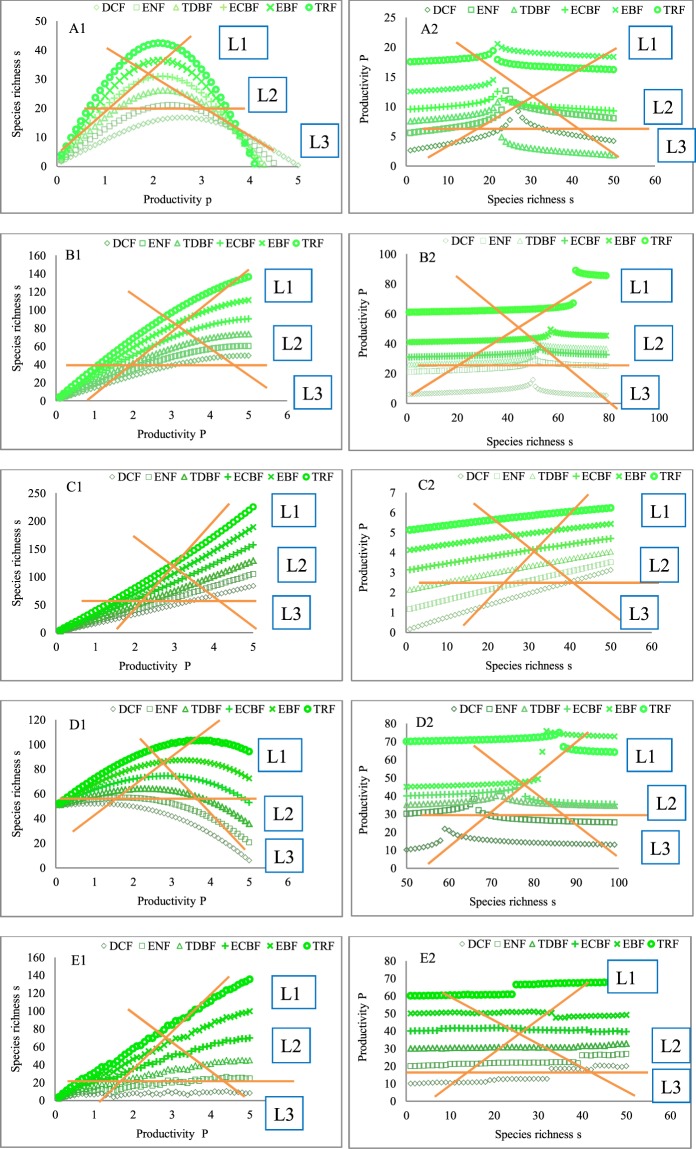
Relationships between the sampling methods and the forms of PSRR and SRPR. L1, L2, and L3 are the three types of sampling methods. Figs A1–E1 and A2–E2 show the five typical forms of PSRR and SRPR arising in the six zonal forests from north to south. Six of the PSRR and SRPR curves in each figure correspond to six sets of the different parameters *m*_1_, *m*_2_, *a*, *E*_h_, *g*, and *k*. In particular, derivation of the five types of PSRR and SRPR starts from DCF, with all parameters assigned the first value in each cell in the data columns marked with # in Supplemental Table 1. Next, successive increments of 0.03, 0.03, 0.1, 5, −0.01, and −0.025 are applied to the values of *m*_1_, *m*_2_, *a*, *E*_h_, *g*, and *k*, respectively, for the next southern forest ENF, TDBF, ECBF, EBF, and TRF. The values of the other parameters remain unchanged. After all the calculations for the forms of PSRR and SRPR using Eqs 10, 11 and 21 in each of the forests were performed, data on the same forms in the six forests were used to produce Figs A1–E1 and A2–E2. Similarly, *P*_0_ and *S*_0_ in a southern forest are considered to be greater than those in a northern forest.

The sampling method also affects the identification of the forms of the PSRR at the regional scale. The local-scale derivation in Fig. 1 indicates that the five typical forms of the PSRR might be present at the local scale in zonal forests, DCF, ENF, TDBF, ECBF, EBF, and TRF in Russia, Japan and the Philippines because of the different strengths of the processes^48^. Moreover, the intrinsic rate of species richness, environmental heterogeneity, resource availability, and species pool size are higher in the southern forests than in the northern ones. Thus, when *m*_1_, *m*_2_, *E*_h_, and *a* in Eqs 10 and 11 are assigned greater parameter values in southern forests compared with those in northern forests, the five typical forms of the PSRR in each of the six forest types are derived. Furthermore, the same forms of the PSRR in the six forests are shown in Fig. 5A1–E1. For the humped forms of the PSRR (Fig. 5A1), if the major quadrats were set up across these zonal forests as described by L1 (i.e., spanning different regions) to gain data on *P* and *s* (i.e., low richness in northern forests corresponding to low plant productivity and high richness in southern forests corresponding to high plant productivity), the form of the PSRR would be the positive form. If the quadrats were set up as described by L2 (i.e., low and high richness versus similar plant productivity), the form of the PSRR would be the irregular form, and if the quadrats were set up as described by L3 (i.e., low richness versus high plant productivity and high richness versus low plant productivity), the form of the PSRR would be the negative form.

As shown in Fig. 5B1–E1, similar results can also be obtained by using a similar set of quadrats. In particular, if the quadrats were set up first as described by L1 and then as described by L2, the form of the PSRR would be the asymptotic form; however, if the quadrats were set up first as described by L1 and then as described by L3, the form of the PSRR would be the humped form. Thus, the forms of the PSRR across these forests (north to south at the regional scale) are diverse and correspond to the various sampling methods. For the SRPR, the application of the L1, L2, and L3 sampling methods allows for the graphical testing of the five SRPR forms (Fig. 5A2–E2) because the different strengths of the processes have also shaped the different SRPRs in these zonal forests.

## Discussion

The forms of the PSRR and SRPR (i.e., the feedback relationships to the PSRR) and the underlying mechanisms are complex and have been intensely debated for several decades^1,3,5,39^. Since ecological processes govern these two relationships through their respective positive and/or negative effects, combining the processes that affect plant species richness and productivity and quantifying their integrative effects is necessary to understand these relationships^39,40^. In this study, we established a set of integration models capable of combining these positive and/or negative processes through differential equations and the dynamic analysis of ecological processes and derived and revealed the various forms of the PSRR and SRPR with variation in the strengths of key processes. The combined processes included the intrinsic rate of species richness (IRSR), resource availability, the species-pool effect, IICE, disturbance, environmental heterogeneity, the SC effect, and density effects. Some of these processes also involve sub-processes^48^. In the real world, these processes regulate the PSRR and SRPR through complex interactions and are not independent factors^39,40,49^. Here, we actually separate them from the complex interaction through specific models before combining them to quantify their roles in the regulation of the PSRR and SRPR and realize a better combination (Methods).

The IRSR is an important process that results in a positive effect of plant productivity on species richness because the speciation rate increases with increasing metabolic rates of plants as well as plant productivity based on the metabolic theory of ecology (MTE)^13^. Moreover, a more productive plant community will possess a high plant density and thus can potentially maintain rare species and high species diversity per species energy theory (SET)^11,12,30^. The integrative models developed here combine the IRSR with the term *m*_1_s in Eq. 1a. Another important term with a positive effect on species richness is *m*_2_*S*_*p*_, which indicates the contribution of immigrant species to species richness; comparatively, the IRSR indicates the contribution of established species to species richness^49,50^. However, with increasing species richness and productivity, IICE increase, which results in a decrease in species richness^21^. We used –*gb* to describe the decrease in Eq. 1a and to establish Eq. 1b to describe the increase in IICE. Here, Eq. 1b is a competing model, and different processes, including the negative effects of increasing productivity, species richness and *S*_p_ on species richness and the negative effects of disturbance on IICE, were combined to derive the different forms of the PSRR and SRPR. Equation 1b produces similar results in terms of shaping the different forms of the PSRR and SRPR due to the combination of processes based on the addition principle in differential calculus. Environmental heterogeneity (*E*_h_) was originally investigated as a classic driver of plant species diversity^20,29^. However, at the local scale, species richness is more dependent on resource availability than on environmental heterogeneity^51,52^. Therefore, we considered the effect of *E*_h_ only at the regional scale in the integration models. Disturbance is a significant factor affecting species richness, productivity and the PSRR, with positive or negative effects^53^. Therefore, in both Eqs 1a and 1b, we introduced a disturbance terms described by the impulse function. All these processes integrated in the models of the PSRR also affect plant productivity; thus, we established models of the SRPR to integrate the effects of these processes and SC effects and density effects on the SRPR.

When ecological processes were assigned different values (Supplemental Table 1), we derived five typical forms of the PSRR at the local scale and indicated the dynamics of IICE and *S*_*p*_ affecting these forms (Fig. 1). The SRPR, i.e., feedbacks to the PSRR, also has five forms (Figs 2 and 3). The SRPR actually includes two types of SRPRs: (1) The SRPR with *s* continually presenting monotonic increases, which has long attracted numerous ecologists to clarify the effects of species richness on plant productivity by conducting manipulative experiments, field investigations, theoretical research and meta-analyses^9,54,55^. The SRRP clearly showed five forms with increasing species richness (*s*) (Fig. 2) when resource availability (*m*_1_ and *m*_2_) were assigned constants (Supplemental Table 1). However, when *m*_1_ and *m*_2_ were assigned increasing values, the five typical forms of the SRRP were not derived (Fig. 4A2–E2), indicating low sensitivity to increasing species richness (*s*) under great variation in resource availability. We suggest that the effect of species richness on productivity is screened by resource availability under a strong condition of resource availability. Similar results have been abundantly observed in the field^4,39,55,56^. (2) The SRPR with *s* continually presenting non-monotonic increases. The derived forms of the SRPR based on complex Eqs 20 and 21 are simply products of coordinate rotation by 90° in Fig. 1A1–E1. The following Figs 4A1–E1 and 5A1–E1 can also be transformed into the SRPR with non-monotonically increasing s after coordinate rotation by 90°. Thus, the SRPR with non-monotonically increasing s can be replaced by the PSRR in this paper. Readers can look at these figures of the forms of the PSRR following coordinate rotation by 90° to understand the forms of the SRPR. For the two SRPR types, the ones with s continually presenting non-monotonic increases are more typical in form than those with *s* continually presenting monotonic increases. Several previous studies have conducted qualitative analyses of some types of possible transformation between the PSRR and SRPR^2,7^. In the present study, the transformation is directly quantified based on integrated models, and the SRPR is classified into the SRPR with *s* continually presenting monotonic and non-monotonic increases.

Abundant empirical data that were compiled over several decades have shown that all of these PSRR and SRPR forms occur in various terrestrial, freshwater, and marine taxa in different regions of the world^1,3,5,39,55^. Ecologists have suggested many theories to explain the underlying mechanisms, but they have been intensely debated^39,40^. The present derivation and model verification performed using observed data clearly indicate that if different strengths of ecological processes are integrated to regulate species richness and productivity (Supplemental Table 1), these five PSRR and SRPR forms will arise (Figs 1 and 2). In particular, when integrated positive processes are dominant, the form of the PSRR and SRPR shows a positively linear or asymptotic pattern; when integrated negative processes are dominant, the form of the PSRR and SRPR shows a negative pattern; and when integrated positive and negative processes are successively dominant, the form of the PSRR and SRPR is humped. The integration models and derived forms of the PSRR and SRPR can almost perfectly explain the observed patterns of the PSRR and SRPR from a dynamical mechanism perspective. The model verification also, to some extent, quantifies the strengths of the processes affecting the observed forms of the PSRR and SRPR^10,38,57–59^. In several reviews of more than 1,000 observational studies conducted in different locations around the world, ecologists have calculated the different ratios of the observed results for each of the five typical forms of the PSRR and SRPR by meta-analysis^1–9,36,39,55^. These studies were conducted in different zonal forests, grasslands and eco-location stations in fields. Ecologists have generally adopted consistent methods for research. Therefore, the observed data are representative and can reflect the observed shapes, and the five typical forms of the PSRR and SRPR are widely accepted by ecologists^1–9,36,39,55^. The present modelling results are also identical to these observed results by comparison of the different forms of the PSRR and SRPR.

Furthermore, intermediate forms of the PSRR and SRPR can also occur (Figs 6 and 7 in Supplemental Material 1) when a community recovers from primary succession with variation in the strengths of key processes. These results suggest that the forms of the PSRR and SRPR can be more diverse than the few typical forms observed in previous studies^5,39^, with the strengths of ecological processes controlling the shape of the PSRR. This is because an increase in the strength of some processes often offsets the strength of other processes and results in the transition to a different form of the PSRR and SRPR. For example, as shown in Fig. 2A1,A2, increasing SC effects and density effects were gradually offset by negative processes (such as IICE and the stress of disturbance) with transitions in *u*(s) and *m*(s) from a rise to a decrease, which consequently shape the peaks of the humped SRPR. Thus, the offset effects of the negative processes on the positive processes can be considered as a mechanism explaining the humped forms of the SRPR. Conversely, in Fig. 4D1,E1, the integration of the different positive processes gradually offset the negative processes, which shape the asymptotic and positive forms of the PSRR. The offset effects of positive processes on negative processes have been investigated in previous empirical studies, in which resource availability offset the negative effects of grazing disturbance in aquatic ecosystems as well as the negative effects of windthrow, logging, or fire in tropical forests on plant diversity^53,60^. These results are very beneficial to the management of ecosystems in terms of coping with climate change and biodiversity decreases in the areas of conservation, forestry and agriculture. Specifically, to maintain high plant diversity and productivity for carbon sequestration and other ecosystem services, the positive processes in Supplemental Table 1 can be considered to be contributory factors to use in the management of ecosystems. When the forms of the PSRR and SRPR are positive and asymptotic, as shown in in Supplemental Table 1, high plant diversity and productivity in communities are able to be well maintained. Correspondingly, the values of the parameters for different processes in the cells corresponding to the positive and asymptotic forms of the PSRR and SRPR can be referred to as a potential standard for the quantitative management of ecosystems. Among these positive processes, the intrinsic rate of species richness, resource availability, the effects of productivity and species richness on intra- and interspecific competition effects and the potential effects of species pools are more important than other processes. Moreover, the values of these processes at moderate levels determine the formation of the positive and asymptotic forms of the PSRR and SRPR. Therefore, ecosystem managers can focus on these key processes.

At the regional scale, we used forests distributed from circumpolar latitudes to the equator as examples and derived the forms of the PSRR and SRPR. Across these zonal forests from north to south, the IRSR, resource availability, the species pool size, and environmental heterogeneity continually increase^61^. Therefore, in the models, the parameters of these processes are assigned greater values for southern forests than for northern forests. Thus, the different forms of the PSRR across these zonal forests change into dominantly positive or close to positive linear forms. However, the SRPR forms often change into a level line, indicating that the sensitivity of productivity to species richness decreases. These results emphasize the importance of the IRSR, resource availability, the species pool, and environmental heterogeneity in the maintenance of plant diversity and productivity from a theoretical perspective, which supports the findings of previous empirical studies^28,52,62,63^. These results indicate that the forms of the PSRR at the regional scale are dictated by dominant positive processes, as observed on different continents, where many positive PSRR gradients in various terrestrial, freshwater, and marine taxa span different latitudes^13^. Notably, different sampling methods also affect the identification of the forms of the PSRR and SRPR at the regional scale, but these sampling methods are only used to reveal the existing PSRRs and SRPRs that are dictated by the strengths of the ecological processes in the real world. These results indicate that both identical and different forms of the PSRR and SRPR occur in plant communities at the regional and local scale. In terms of identical forms, local processes primarily shape the different forms of the PSRR and SRPR at the local scale. The different sampling methods combine the relationships between species richness and productivity occurring at local sampling sites and affected by local processes to create the large-scale forms of PSRR and SRPR spanning different regions. However, in appearance, these large-scale forms of the PSRR and SRPR are identical to the forms occurring at small local scales (Fig. 5). In terms of the different forms, the processes affecting the forms of the PSRR and SRPR across different regions and their strengths differ from those occurring at the local scale. The negative processes, such as intra- and interspecific competition effects, affecting the forms of the PSRR and SRPR and primarily occurring at the local scale are much weaker than the positive processes, such as resource availability and environmental heterogeneity, occurring at the regional scale, which shape the dominantly and regionally positive or asymptotic forms of the PSRR and SRPR^12,57^.

From the perspectives of differential equations and ecological dynamical systems, the integration of these positive or negative processes to derive the different forms of the PSRR and SRPR first suggests that the effects of these processes are identical in nature and, accordingly, that these effects may be accumulated and offset (i.e., there are additive effects and offset effects among ecological processes). When these processes stochastically occur and accumulate or offset the effects of different processes on the PSRR and SRPR, all these processes can create a fluctuant total effect on the forms of the PSRR and SRPR along a gradient of plant productivity or species richness. Thus, the fluctuant total effect may result in the different PSRR and SRPR. Hence, the integration models and derived results are able to explain the recent study results that productivity is a poor predictor of plant species richness^36^. Second, the asymptotic and positive PSRR may change into the humped form if the highest productivity level along a productivity gradient on the x-axis is set to a great value and the PSRR is further modelled. This change occurs because the total effects of the negative processes gradually dominate with increasing productivity or species richness. Fraser *et al*.^8^ published similar findings, in which plant productivity ranged from a low level to a particularly high level and, at the high productivity level, species richness was low because of strong competitive exclusion and resource limitation for some plant species. Thus, the changes in the process strengths described by the integration models may also explain the worldwide (six continents) evidence of a unimodal relationship between productivity and plant species richness^8^.

## Conclusions

The integration methods presented here provide a new theory for interpreting and predicting the results of species richness-plant productivity relationships. The different forms of the PSRR and SRPR are linked to one another under the effects of different processes. The addition of resource availability is known to increase species richness and productivity, and this effect is great at a regional scale in changing the PSRR and SRPR. The ecosystem seems to be unordered because of the actions of different ecological processes; however, when these processes are carefully assessed and combined into a model system that quantifies the integrative roles of these processes, including offset and additive effects, clarifying the PSRR and SRPR in the ecosystem becomes easy. Deriving the forms of the PSRR and SRPR when the mechanisms (i.e., the strengths of the integrated ecological processes determine the PSRR and SRPR forms) operate on different spatial scales would be interesting.

## Methods

### Assessment of combined processes

The combined processes include (1) the intrinsic rate of species richness with increases in plant productivity (IRSR), (2) intra-and interspecific competition effects (IICE), (3) the species-pool effect, (4) disturbance, (5) resource availability, (6) environmental heterogeneity, (7) selection and complementarity effects, and (8) density effects (Supplemental Table 2). Some processes also contained the sub-processes simultaneously combined in the models. The positive or negative effects of all processes and corresponding parameters are given in Supplemental Table 1.

### The models of the PSRR

Theoretically, plant species richness (*s*) continually changes with increases or decreases in plant productivity (*P*) temporally or spatially in communities. This is because *s* is a complex function of various ecological processes (including *P*) that may continually vary in numerical value. Additionally, *s* can be defined in real observations as a positive integer (e.g., 1, 2, and 3^64^). Therefore, we established the following systems to quantify the PSRR, regulated by the aforementioned crucial processes and other processes that structure these crucial processes.

1a $$\frac{ds}{dP}={m}_{1}s+{m}_{2}{S}_{p}-gb-\phi D+\rho {E}_{h}$$

1b $$\frac{db}{dP}=hb+ks+o{S}_{p}-\mu D$$

In Eq. 1a, the term *m*_1_*s* denotes the change in *s* with *P* temporally and spatially in a natural plant community, and *m*_1_ is the intrinsic rate of species richness that increases with *P* (Supplemental Tables 1 and 2). *m*_1_ may also represent the total availability of resources because, according to the MTE and SET, available resources may lead to high metabolic rates, productivity, and gene mutation rates, rapid speciation and more individuals in a plant community, which actually correspond to a high intrinsic rate of species richness^4,11,12,14,19,21,37^. The term *m*_2_*S*_*p*_ reflects increases in *s* when a potential species-pool effect (*S*_*p*_; *s* is the actual effect of the species pool) continues to act on the target community. *m*_2_ represents the total availability of resources, and a high availability may increase the establishment rates of species that immigrated from the species pool. Here, *m*_2_ represents direct availability (e.g., the quantity of soil nutrients), contrasting with indirect availability (*m*_1_), such as plant productivity. *S*_*p*_ is quantified using Eq. 2:

2 $${S}_{{\rm{p}}}=aA-s-lb-E(aA)-\varsigma (aA)-{l}_{1}(aA)$$

The first term, *aA*, is the size of the species pool capable of coexisting in the target community, which is closely related to land area (*A*). *a* is the effect coefficient correlated with geography and climate. The value of *a* is greater when the species pool is located in a relatively humid and hot climatic zone and a complex physiographical region (e.g., a region near the equator), contrasting with species pools located in northern regions. The second term in Eq. 2 is *s*, and with increasing *s* in the target community, the probability of the occurrence of new species immigrating from the species pool decreases; therefore, *S*_p_ will present a decreasing trend with increasing *s*. The term *lb* denotes biotic filtering effects of species in the target community on immigrant species from the species pool through IICE. *E* is the filtering effect coefficient representing the effect of unsuitable abiotic environments on immigrant species in regard to establishment in the target community, and it is also defined as environmental limitations to immigrant species. *ς* is the filtering effect coefficient of several combined factors associated with the limitations of propagation: isolation, dispersal distance, seed fecundity, seed quality and germinability, and dispersal capacity. The filtering effects of various disturbances are characterized by *l*_1_ (*aA*), where *l*_1_ is the effect coefficient. In Eq. 2, *S*_*p*_ ≤ *aA*.

In Equation 1a, *m*_1_*s* and *m*_2_*S*_*p*_ describe a continuous positive increase in *s* with *P*, resource availability, and *S*_p_. However, when *P* and *s* reach high levels, IICE occurs. IICE will weaken the positive increases controlled by *m*_1_*s* and m_2_*S*_*p*_ and even cause a decrease in *s*^16,19,21^. Therefore, we used -*gb* to describe the effect of IICE. *b* represents the effect of IICE given by Eq. 1b, and *g* is the effect coefficient. Disturbance, *D*, which can weaken the positive increases in *s* controlled by *m*_1_*s* and m_2_*S*_*p*_, does not appear at any time. We used an impulse function to describe that it might or might not appear. *E*_h_ represents environmental heterogeneity, which may be assigned different values, even zero, owing to its scale dependence. When *E*_h_ is assigned large values, this indicates high environmental heterogeneity, and consequently *ds/dP* and species richness increase.

In Eq. 1b, *b* monotonically increases with *P*, *s*, and *S*_*p*._ The effects of *P*, *s*, and *S*_*p*_ on *b* may, to a great extent, be independent of one another. The coefficients *h*, *k*, and *o* all are effect coefficients, and their magnitudes determine the contributions of *P*, *s*, and *S*_*p*_ to *b* (Supplemental Table 1). Conversely, *D* in Eq. 1b is able to suppress the dominant species and decrease IICE, which reduces *b* to some extent^60^. Therefore, *μD* is negative, where *μ* is the effect coefficient. In Eqs 1a and 1b, coefficients *m*_1_, *m*_2_, *h*, *k*, and *g* are related to *P*.

After Eq. 2 is substituted into Eqs 1a and 1b, 3 is generated:

3a $$\frac{ds}{d{\rm{P}}}=({m}_{1}-{m}_{2}){\rm{s}}-({m}_{2}l+g)b+{m}_{2}[aA-E(aA)-\varsigma (aA)-{l}_{1}(aA)]-\phi \,D+\rho {E}_{h}$$

3b $$\frac{db}{dP}=(k-{\rm{o}}){\rm{s}}+(h-ol)b+o[aA-E(aA)-\varsigma (aA)-{l}_{1}(aA)]-\mu D$$

The solutions of Eq. 3 exist solely on the basis of the relative judging conditions. After the characteristic roots of the homogeneous form and the particular solutions of Eq. 3 are solved, the general solutions in matrix form are as follows:

4 $$|\begin{array}{c}s\\ b\end{array}|=|\begin{array}{c}{{\rm{C}}}_{1}{{\rm{e}}}^{{{\rm{\lambda }}}_{1}{\rm{P}}}{{\rm{r}}}_{11}+{{\rm{C}}}_{2}{{\rm{e}}}^{{{\rm{\lambda }}}_{2}{\rm{P}}}{{\rm{r}}}_{21}\\ {{\rm{C}}}_{1}{{\rm{e}}}^{{{\rm{\lambda }}}_{1}{\rm{P}}}{{\rm{r}}}_{12}+{{\rm{C}}}_{2}{{\rm{e}}}^{{{\rm{\lambda }}}_{2}{\rm{P}}}{{\rm{r}}}_{22}\end{array}|+\frac{1}{({{\rm{r}}}_{11}{{\rm{r}}}_{22}-{{\rm{r}}}_{12}{{\rm{r}}}_{21})}|\begin{array}{c}\frac{-{{\rm{r}}}_{22}{{\rm{\theta }}e}^{-{{\rm{\lambda }}}_{1}{\rm{P}}}+{{\rm{r}}}_{21}{{\rm{\omega }}e}^{-{{\rm{\lambda }}}_{1}{\rm{P}}}+{{\rm{r}}}_{22}{\rm{\theta }}-{{\rm{r}}}_{21}{\rm{\omega }}}{{{\rm{\lambda }}}_{1}}\\ \frac{{{\rm{r}}}_{12}{{\rm{\theta }}e}^{-{{\rm{\lambda }}}_{2}{\rm{P}}}-{{\rm{r}}}_{11}{{\rm{\omega }}e}^{-{{\rm{\lambda }}}_{2}{\rm{P}}}-{{\rm{r}}}_{12}{\rm{\theta }}+{{\rm{r}}}_{11}{\rm{\omega }}}{{{\rm{\lambda }}}_{2}}\end{array}|$$

Where $\theta ={m}_{2}[aA-E(aA)$ − *ς(aA)* − *l*_1_*(aA*)] − $\phi \,$*D* $+\rho {E}_{h}$; *ω* = *o* $[aA-E(aA)$ − *ς(aA)* − *l*_1_*(aA*)] − *μD;* and *C*_1_ and *C*_2_ are any constants.

5 $${\lambda }_{1}\,{\rm{and}}\,{\lambda }_{2}=\frac{[({m}_{1}-{m}_{2})+(h\mbox{--}ol)]\pm \sqrt{{[({m}_{1}-{m}_{2})+(h\mbox{--}ol)]}^{2}-4\ast 1\ast [(h-ol)({m}_{1}-{m}_{2})+(k-o)({m}_{2}l+g)]}}{2\ast 1}$$

6 $${r}_{11}=\{[(h\mbox{--}ol)-{\lambda }_{1}]-({m}_{2}l+g)\}$$

7 $${r}_{12}=-\,\{[({m}_{1}-{m}_{2})-{\lambda }_{1}]+[(k-o)]\}$$

8 $${r}_{21}=\{[(h\mbox{--}ol)-{\lambda }_{2}]-({m}_{2}l+g)\}$$

9 $${r}_{22}=-\,[({m}_{1}-{m}_{2})-{\lambda }_{2}+(k-o)]$$

Equation 4 (i.e., the solutions to Eq. 3) is very complex, causing the use of this equation to analyse the effects of various ecological processes on the PSRR to be complicated. In addition, the value ranges of the parameters in Eq. 4 that represent the strengths of the ecological processes are highly variable, which results in difficulties in precisely determining their value ranges. Thus, the approximate solutions to Eq. 3 (i.e., Eqs 10 and 11) are given using a fourth-order Runge–Kutta method, a finite difference method. The interception errors of Eqs 10 and 11 are *o*(*h*^5^)^65^. Equations 10 and 11 can directly reflect the change in species richness at any time with various ecological processes. The ecological significance of Eqs 10 and 11 is that the change in current species richness (*s*_i_) and intra- and interspecific competition effects (*b*_i_) is a product of the comprehensive effects of all ecological processes. When *s*_*i*_=0, the dynamics of *s*, *b*, *S*_p_, and *P* from primary succession can be described by Eqs 10 and 11; however, conversely, when *s*_*i*_≠0, the dynamics of *s*, *b*, *S*_p_ and *P* from secondary succession can be evaluated.

10 $${s}_{i+1}={s}_{i}+\delta ({k}_{1s}+2{k}_{2s}+2{k}_{3s}+{k}_{4s})/6$$

11 $${b}_{i+1}={b}_{i}+\delta ({k}_{1b}+2{k}_{2b}+2{k}_{3b}+{k}_{4b})/6$$

In Eq. 10,

12 $${k}_{1s}=({m}_{1}-{m}_{2}){{\rm{s}}}_{{\rm{i}}}-({m}_{2}l+g){b}_{i}+\theta $$

13 $${k}_{2s}=({m}_{1}-{m}_{2})({{\rm{s}}}_{{\rm{i}}}+\delta {k}_{1s}/2)-({m}_{2}l+g)({b}_{i}+\delta {k}_{1b}/2)+\theta $$

14 $${k}_{3s}=({m}_{1}-{m}_{2})({{\rm{s}}}_{{\rm{i}}}+\delta {k}_{2s}/2)-({m}_{2}l+g)({b}_{i}+\delta {k}_{2b}/2)+\theta $$

15 $${k}_{4s}=({m}_{1}-{m}_{2})({{\rm{s}}}_{{\rm{i}}}+\delta {k}_{3s})-({m}_{2}l+g)({b}_{i}+\delta {k}_{3b})+\theta $$

In Eq. 11,

16 $${k}_{1b}=(k-{\rm{o}}){{\rm{s}}}_{{\rm{i}}}+(h-ol){b}_{i}+\omega $$

17 $${k}_{2b}=(k-{\rm{o}})({{\rm{s}}}_{{\rm{i}}}+\delta {k}_{1s}/2)+(h-ol)({b}_{i}+\delta {k}_{1b}/2)+\omega $$

18 $${k}_{3b}=(k-{\rm{o}})({{\rm{s}}}_{{\rm{i}}}+\delta {k}_{2s}/2)+(h-ol)({b}_{i}+\delta {k}_{2b}/2)+\omega $$

19 $${k}_{4b}=(k-{\rm{o}})({{\rm{s}}}_{{\rm{i}}}+\delta {k}_{3s})+(h-ol)({b}_{i}+\delta {k}_{3b})+\omega $$

In Eqs 12–19, s_i_, s_i+1_, b_i_, and b_i+1_ represent s and b at the P_i_ and P_i+1_ levels, respectively.δ is a step value.

### The models of the SRPR

#### Main equation

When the formulae at the left and right sides of Eq. 3a are written in the reciprocal form, the model of the SRPR to describe the feedback relationship to the PSRR is given (Eq. 20). Equation 20 reflects the rate of change in plant productivity with species richness. Equation 20 can be used to test the effects of species richness on plant productivity with the same parameter values, i.e., process strengths, in the derivation of the PSRR by using Eqs 1–19.

20 $$\frac{dP}{ds}=\frac{1}{({m}_{1}-{m}_{2}){\rm{s}}-({m}_{2}l+g)b+{m}_{2}[aA-E(aA)-\varsigma (aA)-{l}_{1}(aA)\,]-\phi D+\rho {E}_{h}}$$

Similarly, the approximate solutions, i.e., Eq. 21, are given to test the feedback effects of species richness on plant productivity and the forms of the SRPR. In the calculation, a set of the same parameter values as those substituted into Eqs 10 and 11 to test the effects of species richness on plant productivity was applied to Eq. 21.

21 $$\,{P}_{i+1}={P}_{i}+\delta ({k}_{1P}+2{k}_{2P}+2{k}_{3P}+{k}_{4P})/6$$

In Eq. 21,

22 $${k}_{1P}=1/[({m}_{1}-{m}_{2}){{\rm{s}}}_{{\rm{i}}}-({m}_{2}l+g){b}_{i}+\theta ]$$

23 $${k}_{2P}=1/[({m}_{1}-{m}_{2})({{\rm{s}}}_{{\rm{i}}}+\delta {k}_{1{\rm{s}}}/2)-({m}_{2}l+g)({b}_{i}+\delta {k}_{1b}/2)+\theta ]$$

24 $${k}_{3P}=1/[({m}_{1}-{m}_{2})({{\rm{s}}}_{{\rm{i}}}+\delta {k}_{2s}/2)-({m}_{2}l+g)({b}_{i}+\delta {k}_{2b}/2)+\theta ]$$

25 $${k}_{4P}=1/[({m}_{1}-{m}_{2})({{\rm{s}}}_{{\rm{i}}}+\delta {k}_{3s})-({m}_{2}l+g)({b}_{i}+\delta {k}_{3b})+\theta ]$$

#### Equations of SC effects and density effects

Furthermore, Eqs 26 and 27 were used to determine the changes in SC effects (*u*(s)) and density effects (*m*(s)) on plant productivity (*P*) with increasing species richness (*s*). At low *s*, in a plant community, increases in temporal or spatial species richness (*s*) result in an increased likelihood of the presence of highly productive species (i.e., selection effects) and the co-occurrence of species through niche partitioning and facilitation (i.e., complementarity effects), which yield positive SC effects on *P*, i.e., an increase in *P* in Eqs 20 or 21 ^9,54^. Therefore, *a*_1_*s* in Eq. 26 is applied to reflect the positive effects of species richness (*s*) on *P*. The coefficient *a*_1_ represents the intensity of the positive effect with increasing *s*. However, when *s* increases to a higher level, *b* begins to increase because plant species with similar niches continually establish and compete for resources^27^. The gradually strengthened *b* weakens increasing *a*_1_*s*, leading to a decrease in *u*(s). *k*_1_*b* in Eq. 26 represents the decreased section of the *u*(s), and *k*_1_ is an effect coefficient of *b*. Disturbance also weakens increasing *a*_1_*s*, and we use *ë*_1_*D* to represent this weakening^39^. *ë*_1_ is the effect coefficient of disturbance. Thus, the effect of *u*(s) on *P* is dictated by the balance among *a*_1_*s*, *k*_1_*b* and *ë*_1_*D* in Eq. 26. In Eq. 26, *a*_1_ is far greater than *k*_1_ at a low level of species richness because an increase in the effects of *b* on *P* is hysteretic temporally or spatially with increasing *s*.

26 $$u(s)={a}_{1}s-{k}_{1}b-{\ddot{e}}_{1}D$$

Meanwhile, plant density also increases with increasing *s* in a plant community based on SET^21,30^. Similarly, no or weak *b* occurs in the plant community when species richness is very low. Thus, the size and mass of individual plants of each plant species are not at all influenced by *b*, which leads to increasing *P* simply because of accumulation in individual mass, i.e., a positive section of the density effect (*m*(s)) on *P*, which can be represented using *a*_2_*s* in Eq. 27. The coefficient *a*_2_ represents the intensity of the effect. However, although increasing diversity results in a greater quantity of individual plants, the average size and mass of individual plants obviously declines at high diversity levels because of strengthened *b*, which decreases *P*. This is the negative section of the density effect (*m*(s)) on *P*, which is represented by *k*_2_*b*. The coefficient *k*_2_ is the intensity of the effect. Disturbance also weakens increasing *a*_2_*s*, and we use *ë*_2_*D* to represent this weakening^60^. *ë*_2_ is the effect coefficient of disturbance. *m*(s) is dictated by the balance among *a*_2_*s*, *k*_2_*b* and *ë*_2_*D*. Similarly, *a*_2_ is far greater than *k*_2_ at a low level of species richness because *b* is hysteretic in effect.

27 $$m(s)={a}_{2}s-{k}_{2}b-{\ddot{e}}_{2}D$$

#### Derivation and verification of the PSRR and SRPR forms

In local ecological communities, it is generally known that species richness is controlled by regional species pools along with species dispersal and ecologically interactive effects, such as competition between species or within species, resource availability, and disturbance^21–23^. Correspondingly, species richness and productivity in the local communities vary^32^. However, many processes occurring at the two scales are different^22^. Therefore, we expected that the forms of the PSRR and SRPR occurring at the local and regional scales would be different. For this comparison, we further developed local and regional derivations of the two relationships to test the theoretical expectations and identify why they would be different.

#### Local scale

The local scale is the spatial extent at which ecological processes occur within the local community. A local community is defined as a set of species that occupy a single relatively homogeneous habitat within a landscape^49,66^. However, if the scale within the local community is further reduced, there also exist different micro-habitats, such as sunny and shady slopes and steep and gentle slopes in a river valley or hills. Therefore, when a series of quadrats or plots are placed in the local community, the productivity levels and availability levels of resources differ among these quadrats or plots^29,51^. Thus, *m*_1_ and *m*_2_ in Eqs 10–25 are considered to be variable values (Supplemental Table 1). At the local scale, environmental heterogeneity (*E*_h_) is actually the level of variation and configuration across various resource types, with diverse configurations and considerable variation resulting in high *E*_h_. A high *E*_h_ likely causes an imbalance in the resource supply for plant species and leads to some plant species being excluded if their critical resource needs are not met^4,27^. Moreover, previous studies have indicated that species diversity is dictated by resource availability rather than environmental heterogeneity at the local scale^27,28^. Therefore, the *E*_h_ among these quadrats or plots at the local scale is assigned to be zero in Eqs 10–19. With respect to coefficients *g*, *h*, and *k*, they increase with increasing *P* and *s*, and they consequently also take different values (Supplemental Table 1). *O*, *a*, *A*, *ς*, *E*, *l*_1_, *a*_1_, *a*_2_, *k*_1_, and *k*_2_ might be constants because the PSRR and SRPR at the local scale are derived assuming a deterministic target community affected by a deterministic species pool. Specifically, the least-squares method and the stochastic approximation method were used to estimate the values of the parameters of Eqs 10, 11 and 21 (i.e., the solutions of Eqs 1 or 3 and 20) by substituting different parameter values into Eqs 10, 11 and 21 to test the forms for the PSRR and SRPR. Finally, five sets of the parameter values that can be used to derive the five typical forms of the PSRR and SRPR were determined (the first value in each cell in the data columns with # in Supplemental Table 1). For these parameters, the greater the value, the stronger the ecological process that is represented, and we used dimensionless units.

To verify the derived forms of the PSRR and SRPR, we used the observed data of ten classic PSRRs and SRPRs to estimate the parameter values of Eqs 10, 11 and 21, i.e., the second and third values in each cell in the data columns with # in Supplemental Table 1^10,38,56–59,67,68^. Next, these parameter values were substituted into Eqs 10, 11 and 21 to derive the PSRR and SRPR forms. The differences between the derived and observed PSRR and SRPR forms were tested using *t*-tests and goodness-of-fit tests. The details are shown in Supplemental Table 1.

We also assessed the responsive changes of the PSRR and SRPR forms to different strengths of two key processes, resource availability and intensity of IICE, when ecosystem restoration temporally and spatially begins from primary succession (Supplemental Material 1). In such cases, *s*_*i*_ = 0 in Eqs 10, 11 and 21, and there are no propagules in the ecosystem. Accordingly, *S*_p_ has a particular significance because the potential species-pool effect and availability levels of resources, which affect the rates of species establishment, are crucial positive processes that increase *s* as well as *P*^50^.

#### Regional scale

The PSRR at the regional scale was defined as the relationship between *P* and *s* across different local communities within a metacommunity or in different biogeographical provinces^66^. A metacommunity is a set of local communities linked by the dispersal of multiple interacting plant species within a landscape or region, and these local communities often occur in different habitats, such as valleys, hills, river beaches, wetlands, and deltas. Habitat heterogeneity (i.e., environmental heterogeneity at the local scale) is known to differ among local communities and strongly drives the variation in *s* and *P* according to the heterogeneity-diversity hypothesis^20,66^. Therefore, variable values are required for *E*_h_ in Eqs 1–27. The availability levels of rsources are also variable among these local communities; therefore, *m*_1_ and *m*_2_ in these equations also need to be assigned different values. For local communities in various biogeographical provinces, they are distributed across various latitude belts, such as tropical or temperate zones, with different climatic types and geographical characteristics. The variation in *E*_h_, *m*_1_, and *m*_2_ is high.

We assumed that a long transect is located in the Northern Hemisphere across deciduous coniferous forests (DCF), evergreen needle-leaf forests (ENF), temperate deciduous broad-leaved forests (TDBF), evergreen coniferous and broad-leaved mixed forests (ECBF), evergreen broad-leaved forests (EBF), and tropical rainforests (TRF) from circumpolar latitudes to the equator. Clearly, along this transect, there is a gradual increase in *P* and resource availability, such as the annual mean temperature and precipitation; thus, mineral nutrients are relatively richer in southern forests than in northern forests^13,61^. Accordingly, *m*_1_ and *m*_2_ in Eqs 10, 11 and 21 were assigned greater values to derive the forms of the PSRR and SRPR in southern forests. For *E*_h_, topographical variability and surface roughness are two indices that accurately describe the *E*_h_ at the landscape and regional scales. With increasing topographical variability and surface roughness, variability in habitat types and their diverse spatial configurations, as well as variation in the soil environment, will increase, and this will result in high species diversity^51,52,62,63^. In comparison, topographical variability and surface roughness in northern forests are lower because of the relatively gentle topography and small variation in elevation due to historical geological reasons and weaker rainfall erosion^69,70^. Therefore, *E*_h_ values are greater for southern forests than for northern forests. In addition, the speciation rate increases from the north to south based on MTE; consequently, a high number of species accumulate over evolutionary time in southern forests with the same area (A)^14^. Therefore, the size of the species pools (*aA*) is greater in the south than in the north, and *a* in these equations gradually increases from north to south^13,14,60^. However, other parameters show small variation; therefore, their values were assumed to be invariable like those used to derive Fig. 1A1–E1 by using the values in Supplemental Table 1.

## Supplementary information

Supplemental Table 1

Supplemental Table 2

Supplemental Mateiral 1

## Data Availability

The datasets generated during and/or analysed during the current study are available from the corresponding author on reasonable request.
