# Supplementary material for: Integrative models explain the relationships between species richness and productivity in plant communities
Source: Sci Rep. 2019 Sep 24;9:13730. doi: 10.1038/s41598-019-50016-3 (PMC6760178; doi:10.1038/s41598-019-50016-3)
Supplement: Supplementary file 1 — Supplemental Table 1 [file 41598_2019_50016_MOESM1_ESM.pdf]

Integrative models explain the relationships between species richness and productivity in plant communities

Zhenhong Wang[1], Alessandro Chiarucci[2], Juan F. Arratia[3]

[1]Key Laboratory of Subsurface Hydrology and Ecological Effects in Arid Regions, Ministry of Education, Chang`an University, Xi`an, China;

School of Environmental Science and Engineering, Chang`an University, Xian 710064, China

[2]Department of Biological, Geological and Environmental Science, University of Bologna, Via Irnerio 42-40126, Bologna, Italy

[3]AGMUS Institute of Mathematics, Caribbean Computing Center for Excellence, 21150, San Juan, Puerto Rico, USA

Correspondence: E-mail: w_zhenhong@126.com

Supplemental Table 1: Variables and parameters in models 1–27.

Symbol	Ecological processes	The effects of ecological processes on species richness: positive (+), negative (-) or no any (0) effects, and citations	The assigned parameter values corresponding to five typical forms of PSRR and SRPR				
			Humped form#	Asymptotic form#	Positive form#	Negative form#	Irregular form#
m_1	The intrinsic rate of species richness that increases with plant productivity	(+)(Steven & Carson1999, Cardinale et al. 2009, Stegen Enquist and Ferriere 2009, Adler et al. 2011)	0.10, **0.20, 0.70**	0.13 **0.20, 0.05**	0.25, **0.12,0.15**	0.05, **0.02, 0.20**	0.33, **0.10,**0.33
m_2	* Resource availability	(+ or -)(Baer et al. 2004, Cardinale et al. 2009,Gundale et al. 2011; Stevens and Carson.2002)	0.15 **0.20, 0.40**	0.23, **0.11, 0.03**	0.25, **0.20, 0.30**	0.15, **0.01,** 0.15	0.25, 0.25,0.25
h	Effect coefficient of productivity on IICE	(-) (Goldbergand and Werner 1983, Huston and	0.18	0.25,	0.08,	0.35,	0.20,

	(intra-and inter-specific competition effects)	DeAngelis 1994)	0.18, 0.18	**0.10, 0.05**	0.08, 0.08	**0.60,** 0.35	0.20, 0.20
k	Effect coefficient of species richness on IICE	(-)(Newman 1973, Tilman 1982,Goldberg and Miller 1990)	0.90,<br>**0.50,0.75**	0.22,<br>**0.28, 0.25**	0.10,<br>**0.20, 0.08**	0.28,<br>**0.65, 0.40**	0.20,<br>0.20, 0.20
l	IICE coefficient of established species on immigrating species	(-)(Newman 1973, Keddy1992, Pärtel et al.1996, Zobel et al. 1998)	0.70<br>**0.65, 0.20**	0.45，<br>**0.25, 0.05**	0.10,<br>**0.15**, 0.10	0.10,<br>**0.70**, 0.10	0.45,<br>0.45, 0.45
g	IICE coefficient on species richness	(-)(Newman 1973, Tilman 1982,Goldberg and Miller 1990,Steven and Carson 1999)	0.60,<br>**0.55, 0.30**	0.55，<br>**0.15, 0.30**	0.40,<br>**0.35, 0.05**	0.30,<br>**0.80, 0.20**	0.38,<br>0.38, **0.38**
a	Coefficient of the species-pool effect across different biogeographical provinces	(+)(Keddy 1992, Pärtel et al.1996, Zobel et al. 1998)	1.00,<br>1.00, 1.00	1.00<br>1.00, 1.00	1.00,<br>1.00, 1.00	1.00,<br>1.00, 1.00	1.00,<br>1.00,1.00
o	Effect coefficient of S_p (potential species-pool effect) on IICE	(-)(Keddy 1992, Pärtel et al.1996, Zobel et al. 1998)	0.04,<br>0.04, 0.04	0.02,<br>0.02, 0.02	0.02,<br>0.02, 0.02	0.02,<br>0.02, 0.02	0.02,<br>0.02, 0.02
E	Coefficient of filtering effect of the unsuitable environment on immigrating species	(-)(Rajaniemi et al. 2006, Myers and Harms 2009)	0.20,<br>0.20, 0.20	0.20,<br>0.20, 0.20	0.20,<br>0.20, 0.20	0.20,<br>0.20, 0.20	0.20,<br>0.20, 0.20
ς	Coefficient of filtering effect of dispersal limitation on immigrating species	(-)(Rajaniemi et al. 2006, Myers and Harms 2009)	0.10,<br>0.10, 0.10	0.10,<br>0.10, 0.10	0.10,<br>0.10, 0.10	0.10,<br>0.10, 0.10	0.10,<br>0.10, 0.10
A	Land area	(+)(Keddy 1992, Pärtel et al. 1996)	100,<br>100,100	100,<br>100, 100	100,<br>100, 100	100,<br>100, 100	100,<br>100,100
D	*Disturbance intensity	(- or +) (Belsky 1992, Matsinos and Troumbis 2002)	100,<br>100, 100	100<br>100,100	100,<br>100, 100	100,<br>100, 100	1-1000,<br>**60-567,**1-600
μ	Effect coefficient of disturbance on IICE	(+) (Matsinos and Troumbis 2002, Zunzunegui et al. 2012)	0.080,<br>**0.05**, 0.08	0.05,<br>0.05, 0.05	0.05,<br>0.05, 0.05	0.05,<br>0.05, 0.05	0.05,<br>0.05, 0.05
φ	Effect coefficient of disturbance on species richness	(-)(Belsky 1992, Deng et al. 2014)	0.01,<br>0.01, 0.01	0.01,<br>0.01, 0.01	0.01,<br>0.01, 0.10	0.01,<br>0.01, 0.10	0.1,<br>0.10,0.10

l_1	Effect coefficient of disturbance on S_p	(-)(Keddy1992, Pärtel et al. 1996, Zobel et al.1998)	0.14, 0.14, 0.14	0.14, 0.14, 0.14	0.14, 0.14, 0.14	0.14, 0.14, 0.14	0.14, 0.14, 0.14
ρ	*Effect coefficient of environmental heterogeneity on species richness	(0 or +) (Nichols et al. 1998, Lundholm and Larson 2003, Baer et al. 2004, Dufour et al. 2006)	0，0, 0	0，0, 0	0，0, 0	0，0, 0	0，0, 0
E_h	*Environmental heterogeneity	(0 or +)(Baer et al. 2004, Reynolds and Haubensak 2008, Gundale et al. 2011)	0，0, 0	0，0, 0	0，0, 0	0，0, 0	0，0, 0
ΔP	*Increment of plant productivity	(+ or -)(Steven and Carson1999, Huston 2014)	0.10, 0.10, 0.10	0.10, **0.033, 0.048**	0.10, 0.10, 0.10	0.10, **0.014, 0.30**	0.10 0.021,0.10
Δs	*Increment of species richness	(+ or -)(Loreau et al. 2001; Balvanera et al. 2006)	1, -, **0.54**	1, -,**0.60**	1,-,**0.28**	1,-,**0.23**	1，-,**0.04**
s_0	*Initial species richness	(- or +)(Tilman 1982, Collinson 1978)	1, **14, 1**	1, **22,**,1	1, **22**, 1	50, **19**, 50	0 **4，0**
b_0	Initial IICE	(-)(Newman 1973, Goldberg and Miller 1990)	0.10, 0.10, 0.10	0.10, 0.10, 0.10	0.10, 0.10, 0.10	0.20, 0.20, 0.20	0.00 **0.10**, 0.00
P_0	*Initial plant productivity	(+ or -)(Adler et al. 2011,Rees 2013)	2.50 **-, 0.75**	1.00, **-, 0.00**	0.10 **-, 6.25**	10, **-, 6.30**	10 **-,5.8**5
a_1	Effect coefficient of specie richness on SC effect	(+)(Loreau et al. 2001; Balvanera et al. 2006)	0.11	0.11	0.11	0.11	0.11
a_2	Effect coefficient of specie richness on density effect	(+)(Srivastava and Lawton 1998; Stevens and Carson 1999)	0.165	0.165	0.165	0.165	0.165
k_1	Effect coefficient of IICE on SC effect	(-)(Tilman 1982; Balvanera et al. 2006)	0.07	0.07	0.07	0.07	0.07
k_2	Effect coefficient of IICE on SC effect	(-)(Huston and DeAngelis 1994; Srivastava and Lawton 1998)	0.10	0.10	0.10	0.10	0.10
$\ddot{e}_1$	Effect coefficient of disturbance on SC effect	(-)(Grace et al. 2014; Deng et al. 2014)	0	0	0	0	0.007
$\ddot{e}_2$	Effect coefficient of disturbance on density	(-)(Belsky 1992; Bongers et al. 2009)	0	0	0	0	0.01

	effect						
s	*Species richness	(- or +)(Tilman 1982, Collinson 1978)	Dependent variable				
P	*Plant productivity	(+ or -)(Goldberg and Werner 1983, Huston and DeAngelis 1994, Steven and Carson1999, Cardinale et al. 2009)	Independent variable				
b	IICE	(-)(Newman 1973, Tilman 1982, Goldberg and Miller 1990, Steven and Carson 1999)	Dependent or independent variable				
S_p	Potential species-pool effect	(+) (Keddy 1992, Pärtel et al.1996)	Dependent or independent variable				
u(s)	Sampling and complementarity (SC) effect	(+)(Loreau et al. 2001; Balvanera et al. 2006)	Dependent or independent variable				
m(s)	Density effect	(+)(Srivastava and Lawton 1998; Stevens and Carson 1999)	Dependent or independent variable				

Notes: Processes marked with an asterisk are those that have several effects. For example, m_2 has remarkable positive effects on species richness at low productivity levels, but its effect might be negative at high productivity levels, which could result in a negative *ds/dP* in equation 1a and a decrease of *s*.

The first value in each cell in the data columns marked with # is estimated using the stochastic approximation method (Robbins and Monro1951).

The second value in each cell in the data columns marked with # is estimated using both the least-squares method and stochastic approximation method based on the observed *s* and *P* of the five typical forms of PSRR at local scales in the forests of Germany (humped), Russia (positive), and Czech Republic+Germany+Russia (asymptotic; Axmanová et al. 2012); in a Kansas grassland of the USA (negative; Foster et al. 2004); and a woodland of New South Wales in Australia (irregular; Allcock and Hik 2003). Different levels of disturbance were noted in the woodland in New South Wales, owing to a high abundance of kangaroos and rabbits and human activities.

The third value in each cell in the data columns marked with # is also estimated using the least-squares method and stochastic approximation method based on the observed *s* and *P* in experimental grasslands of Texas (humped; Grace et al. 2007), the forest plots across the world (asymptotic; Chisholm et al. 2013), the floodplain of the Saale River in Germany (positive; Marquard et al. 2009), plant community in Gloucestershire of the UK (negative; Thompson et al. 2005), and the Czech Republic (irregular; Rychtecká et al. 2004). Further, disturbance for SRPR was noted in the Czech Republic. These estimated values, to some extent, reflect the strengths of the processes shaping the forms of PSRR and SRPR in these observations. Values presented in bold are different from the first estimated value.

References (excluded in the reference list of main text)

1. Belsky,A.J.1992. Effects of grazing, competition, disturbance and fire on species composition and diversity in grassland communities. Journal of Vegetation Science 3: 187–200.
2. Cardinale, B.J., D.M. Bennett, C.E.Nelson, and L.Gross.2009. Does productivity drive diversity or vice versa? A test of the multivariate

productivity–diversity hypothesis in streams. Ecology 90: 1227-1241.
3. Deng, L., S.Sweeney, and Z.P.Shangguan.2014. Grassland responses to grazing disturbance: plant diversity changes with grazing intensity in a desert steppe. Grass and forage science 69: 524–533.
4. Goldberg, D. E., and T. E. Miller. 1990. Effects of different resource additions on species diversity in an annual plant community. Ecology 71: 213-225.
5. Grace,J.B., T.M. Anderson, M.D. Smith, E.Seabloom, S.J. Andelman, G.Meche, et al.2007. Does species diversity limit productivity in natural grassland communities? Ecology Letters 10: 680–689.
6. Gundale, M.J., A.Fajardo, R.W. Lucas, M.C.Nilsson, and D.A.Wardle.2011. Resource heterogeneity does not explain the diversity-productivity relationship across a boreal island fertility gradient. Ecography 34: 887-896.
7. Keddy, P. A. 1992. Assembly and response rules: Two goals for predictive community ecology. Jouranl of Vegetation Science. 3,157-164.
8. Loreau, M., A. Hector. 2001. Partitioning selection and complementarity in biodiversity experiments. Nature 412: 72-76.
9.
10. Myers, J.A., and K.E.Harms. 2009. Seed arrival, ecological filters, and plant species richness: a meta-analysis. Ecology Letters 12: 1250–1260.
11. Rajaniemi, T. K., D. E.Goldberg, R. Turkington, and A. R.Dyer. 2006. Quantitative partitioning of regional and local processes shaping regional diversity patterns. Ecology Letters 9: 121-128.
12. Rees, M .2013. Competition on productivity gradients—what do we expect? Ecology Letters 16: 291–298.
13. Robbins, H., and S. Monro. 1951. A Stochastic approximation method.Annals of Mathematical Statistics 22:400-4074.
14. Stevens, M.H.H., and W.P. Carson.2002. Resource quantity, not resource heterogeneity, maintains plant diversity. Ecology Letters 5: 420-426.
