# Supplementary material for: Integrative models explain the relationships between species richness and productivity in plant communities
Source: Sci Rep. 2019 Sep 24;9:13730. doi: 10.1038/s41598-019-50016-3 (PMC6760178; doi:10.1038/s41598-019-50016-3)
Supplement: Supplementary file 2 — Supplemental Table 2 [file 41598_2019_50016_MOESM2_ESM.pdf]

Integrative models explain the relationships between species richness and productivity in plant communities

Zhenhong Wang[1], Alessandro Chiarucci[2], Juan F. Arratia[3]

[1] Key Laboratory of Subsurface Hydrology and Ecological Effects in Arid Regions, Ministry of Education, Chang`an University, Xi`an, China;

School of Environmental Science and Engineering, Chang`an University, Xian 710064, China

[2]Department of Biological, Geological and Environmental Science, University of Bologna, Via Irnerio 42-40126, Bologna, Italy

[3]AGMUS Institute of Mathematics, Caribbean Computing Center for Excellence, 21150, San Juan, Puerto Rico, USA

Supplemental Table 2 Primary processes that were combined in models

Ecological processes	Explanation of the causes why the ecological process was introduced in models
(1) Intrinsic rate of species richness with increases in plant productivity.	Intrinsic rate of species richness (IRSR) is defined as a positive process regulating plant species richness, productivity, and PSRR according to the metabolic theory of ecology (MTE) and species energy theory (SET). MTE states that available resources such as high ambient temperature and precipitation can generate a high metabolic rate in plants from the biochemical kinetics of metabolism and productivity; the high metabolic rate increases the mutation rate of genes and causes rapid speciation, resulting in higher species richness in communities of warmer and more humid regions than that of relatively colder and drier regions (Stegen et al. 2009, Adler et al. 2011). Thus, the species richness increase is inherent to increasing productivity. SET explains that species richness tends to increase with increasing productivity of a community or the increasing humped portion of PSRR (Wright 1983, Wright et al. 1993, Cardinale et al. 2009). SET assumes that a certain

	number of resources is required to maintain at least one individual of a given species; therefore, as the quantity of resources (i.e., potential productivity) increases, the quantity of individuals increases, and more rare species are also able to maintain viable population sizes in a community, which leads to reduced local extinction and increased species diversity (Wright 1983, Wright et al. 1993). SET is based on the premise that increases in the number of individuals and productivity are positively correlated, and, consequently, productivity is simply a sum of individual growth; these actually occur only when there is low resource availability or productivity levels with weak or no competition stress (Fish et al. 1943, Jiang et al. 2002, Cook-Patton et al. 2011). Srivastava and Lawton (1998) even referred to this as the more-individuals hypothesis.
(2) Intra-and inter-specific competition effects.	The intra- and inter-specific competition effects (IICE) include multiple processes such as competition stress, inter-specific competitive exclusion (ICE), and assemblage-level thinning (ALT), and these processes are not mutually exclutive (Goldberg and Miller1990, Huston and DeAngelis 1994). Most competition theories indicate that mortality is not equal among plant species, and that ICE reduces species richness in habitats with abundant resources and high plant productivity (Steven and Carson 1999). In particular, differences between species for resource use, individual size, and growth rates create competitive hierarchies. Competition for limiting resources becomes increasingly important at high levels of plant productivity; thus, resource acquisition and growth by the dominant species suppresses and, eventually, excludes the subordinate species, thereby yielding a relatively species-poor assemblage (Goldberg and Werner 1983, Huston and DeAngelis 1994, Steven and Carson 1999). ALT occurs because of equal mortality among plant species and thinning alone in habitats with abundant resources, and high plant productivity causes decreases in species richness, leading to the extinction of rare species (Tilman and Pacala 1993, Wright et al.1993, Abrams1995, Oksanen 1996). ICE and ALT both explain decreases in plant species richness (i.e., a negative effect on species richness) for high resource availability levels. However, ICE and ALT often have positive effects on plant productivity in a community when they eliminate subordinate species or weak individuals in competition for resources, but competition stress without species excluded may weaken the growth of all species, generating a negative effect on plant productivity (Newman 1973, Tilman 1982, Steven and Carson 1999). Clearly, IICE on species richness and productivity are complex.
(3)Species-pool effect.	Species pools are defined as a set of plant species with each species of a community, local, or regional flora being a member of any community, local, or regional species pool, with different degrees of probability. The probability is specifically regulated in a plant species assembly by the ecological and limiting similarities and differences of niches, fitness, and competitive ability occurring among, for example, specific plant species, diaspore abundance, dispersal distance, dispersal capacity, dispersal mechanisms, germinability of newly arrived diaspores, and longevity of seeds in the diaspore bank (Taylor et al.1990, Zobel et al.1998, Mayfield and Levine 2010, Hillerislambers et al. 2012). Species richness occurring

	on a particular scale, as well as the geographical extent of that scale, is dictated by species-pool effect (i.e., the actual contribution of species pool to species sources). Species-pool effect is weakened owing to filtering by abiotic and biotic processes on immigrating species (Keddy 1992, Pärtel and Zobel 19968).For example, isolation, long-dispersal distance, unsuitable establishment conditions, lack of seeds, and differences in fitness and competitive ability between immigrated and established species cause decreases in the species-pool effect; therefore, these factors have a negative effect on species richness (Mayfield and Levine 2010). Species-pool effect is also closely related to geographical and evolutionary contexts, in that, species pool, delimited by a geographical region with a large land area, high environmental heterogeneity, resource abundance, and rapid speciation, or a region in the center of biodiversity, possesses a greater species-pool effect (Zobel1992).
(4) Disturbances.	Intense disturbances by natural or human factors, such as grazing, fire, severe windstorms, wave damage, land cover alterations, habitat fragments, and forest destruction, often change plant productivity and species richness, primarily via a negative mortality-causing effect, and further regulate PSRR and SRPR (Matsinos and Troumbis 2002, Hughes et al. 2007, Nuttle et al. 2013). However, moderate disturbances such as a moderate intensity of grazing and fires often restrain dominant plants and remove litter and secondary biomass in forests, promoting the establishment of immigrated species, which in turn results in an increase in plant diversity and productivity—an indirect effect (Belsky 1992,Zunzunegui et al. 2012). Consequently, the “intermediate disturbance hypothesis” has been widely recognized as an ideal concept for explaining the effects of disturbances on species diversity patterns, although recent critiques have recommended rejecting the famous hypothesis because of some theoretical weaknesses (Belsky 1992, Nuttle et al. 2013, Huston 2014). Therefore, in integration models, disturbance is introduced as a dominant negative effect, but a positive indirect effect is also considered.
(5) Resource availability.	Resource availability includes two characteristics. First, total abundance of each resource, such as temperature, sunlight, water, and mineral nutrients directly affects plant species richness and productivity by controlling sizes of populations, growth of organisms, and the probability of stochastic extinction according to SET (Baer et al. 2004, Cardinale et al. 2009). Second, relatively more quantities in limiting resources ensure that weaker competitors are able to capture limiting resources not being consumed, based on the resource ratio theory. The theory states that imbalance in the supply of two or more resources, relative to the stoichiometric needs of competitors, can dictate the strength of competition and, in turn, the diversity of coexisting species (Tilman 1982, Cardinale et al. 2009,Gundale et al. 2011). Therefore, a balanced resource supply or high resource availability can help maintain species coexistence.
(6) Environmental heterogeneity.	Environmental heterogeneity is scale-dependent, and, at a local-scale level (e.g., quadrats and plots), there are different configurations in resource types with various availability levels along with more complex configurations in abiotic and biotic resources and environmental heterogeneities (Baer et al. 2004, Reynolds and Haubensak 2008). For example, five levels of

	pH, depth, moisture, and nitrogen (i.e., four factors) in the soil are configured in a plot, and accordingly, environmental heterogeneity in the plot will exceed another plot in which only four levels of these four factors or five levels of three factors are configured. However, environmental heterogeneity primarily embodies differences in habitat and microclimate at landscape scales, such as the habitat differences of the sites when plant communities are located within a landscape (Nichols et al. 1998, Lundholm and Larson 2003, Dufour et al. 2006).Therefore, a set of relatively different species arises in different habitats, which increases species diversity. The climate and physiography of biogeographical provinces present obvious differences on regional scales, which shape various zonal vegetation types (Song 2001, Lasky et al. 2014). Higher heterogeneity in climate and physiography is noted for southern regions (especially areas near the equator) than for northern regions. This is because of the relatively complete vertical climate belts and formations of topography (Kim et al. 2000, Sun and Zhang 2013).
(7) Selection and complementarity (SC) *effects.*	Selection effect is the standard statistical covariance effect, and specifically, total productivity in a plant community is on average higher than that of the weighted average based on monoculture of the component species, because a diverse community stochastically contains highly productive species (Loreau and Hector 2001, Balvanera et al. 2006). Complementarity effect actually refers to an effect caused by differentiation in resource use and/or facilitative interactions among species, which become the main drivers of increasing productivity at higher levels of species richness (Balvanera et al. 2006, Cardinale et al. 2007). However, selection effect is challenged by the so-called zero-sum game, which states that, in a diverse community, the low productive species also occur at a high probability and offset the effect of highly productive species, thereby reducing the effect to zero (Cardinale et al. 2007,Turnbull et al. 2013). Thus, in this model, we considered the two effects as one integral selection and complementarity (SC) effect. The SC effects primarily enhance the mass of individual plants to increase plant productivity, which is similar to *k* selection in plant strategy.
(8) Density effects.	Density effects are based on *species-energy theory*, which suggests positive relationships between species richness and total number of plants in plant communities (Wright et al. 1993, Srivastava and Lawton 1998). In particular, plant density increases with increasing species richness, thus leading to high biomass production at low inter-specific and intra-specific competition levels, an effect that appears to have been previously ignored (Stevens and Carson 1999, Cook-Patton et al. 2011). However, inter-specific competitive stress on the growth of plants occurs at high species richness levels to cause a decrease of the average size and mass of individual plants, and thus plant productivity (Tilman 1982, Goldberg and Werner 1983, Huston and DeAngelis 1994). Density effects reflect a characteristic of plant species richness by increasing the density of plants to increase plant productivity, and it is similar to *r* selection in plant strategy.

References (Excluded in the reference list of main text)

1. Cardinale, B.J., D.M. Bennett, C.E.Nelson, and L.Gross.2009. Does productivity drive diversity or vice versa? A test of the multivariate productivity–diversity hypothesis in streams. Ecology 90: 1227-1241.
2. Cook-Patton, S.C., S.H.Mcart, A. L.Parachnowithsch, J.S.Thaler, and A.A.Agrawal.2011.A direct comparison of the consequences of plant genotypic and species diversity on communities and ecosystem function. Ecology 92: 915-923.
3. Fish, R.A., A.S.Corbet, and C.B. Williams.1943. The relationship between the number of species and the number of individuals in a random sample of an animal population. Journal of Animal Ecology 12: 42-58.
4. Goldberg, D. E., and T. E. Miller. 1990. Effects of different resource additions on species diversity in an annual plant community. Ecology 71: 213-225.
5. Gundale, M.J., A.Fajardo, R.W. Lucas, M.C.Nilsson, and D.A.Wardle.2011. Resource heterogeneity does not explain the diversity-productivity relationship across a boreal island fertility gradient. Ecography 34: 887-896.
6. Hillerislambers, J., P.B. Adler, W.S.Harpole, J.M. Levine, and M.M.Mayfield. 2012. Rethinking community assembly through the lens of coexistence Theory. Annual Review of Ecology, Evolution & Systematics 43: 227–48.
7. Hutchinson, G.E. 1959. Homage to Santa Rosalia, or why are there so many kinds of animals? The American Naturalist93: 145–159.
8. Jiang, Y. X., B.S.Wang, R.G.Zang, J.H.Jin, and W.B Liao.2002. Biodiversity and Formation Mechanism of Tropical Forest in Hainan Island in China. Science Press, Beijing, PRC.
9. Mayfield,M.M, andJ.M.Levine. 2010. Opposing effects of competitive exclusion on the phylogenetic structure of communities. Ecology Letters 13: 1085–1093.
10. Song,Y.C. 2001. Vegetation Ecology. Higher Education Press, Beijing, PRC.
11. Turnbull, L.A., J.M. Levine,M. Loreau, and A.Hector. 2013. Coexistence, niches and biodiversity effects on ecosystem Functioning. Ecology Letters 16: 116-127.
12. Zunzunegui, M., M.P.Esquivias, F.Oppo, and J.B.Gallego-Fernández.2012. Interspecific competition and livestock disturbance control the spatial patterns of two coastal dune shrubs. Plant and Soil 354: 299–309.
