# Supplementary material for: Integrative models explain the relationships between species richness and productivity in plant communities
Source: Sci Rep. 2019 Sep 24;9:13730. doi: 10.1038/s41598-019-50016-3 (PMC6760178; doi:10.1038/s41598-019-50016-3)
Supplement: Supplementary file 3 — Supplemental Mateiral 1 [file 41598_2019_50016_MOESM3_ESM.pdf]

Integrative models explain the relationships between species richness and productivity in plant communities

Zhenhong Wang[1*], Alessandro Chiarucci[2], Juan F. Arratia[3]

[1]Key Laboratory of Subsurface Hydrology and Ecological Effects in Arid Regions, Ministry of Education, Chang`an University, Xi`an, China; School of Environmental Science and Engineering, Chang`an University, Xian 710064, China

[2]Department of Biological, Geological and Environmental Science, University of Bologna, Via Irnerio 42-40126, Bologna, Italy

[3]AGMUS Institute of Mathematics, Caribbean Computing Center for Excellence, 21150, San Juan, Puerto Rico, USA

Correspondence: E-mail: w_zhenhong@126.com

Supplemental Material 1

The responsive changes of PSRR and SRPR

When resource availability m_2 is assigned a greater value than 0.15, the peak value of s for the humped curve of PSRR at the level of 0.15 resource availability increases, and the s-peak occurs at a small P level (Fig.6 A1). Conversely, when resource availability m_2 is assigned a smaller value than 0.15, the humped curve of PSRR at the level of 0.15 resource availability changes into the asymptotic form, and the s-peak occurs at a high P level (Fig.6 A1). However, increasing m_2 and s also gradually increase intra-and inter-specific competition effects b (Fig.6 A2), and consequently, s rapidly decreases after s has reached its peak value (Fig.6 A1). When IICE (intra-and inter-specific competition effects) coefficient g is assigned gradually increasing values, the asymptotic form of PSRR shows a gradual transition from an asymptotic to humped form, and a large vale of IICE coefficient g leads to the humped form of PSRR (Fig.6 A3). However, the difference in b among curves is small (Fig.6 A4). The asymptotic and positive forms of PSRR and the corresponding dynamics of b show similar variations with changes in m_2 and g (Fig.6 B1-B4, Fig.6 C1-C4). In particular, when m_2 is very small, the two types show a positive form of PSRR; when m_2 becomes larger, the positive form gradually changes into an asymptotic form or similar to the humped form (Fig.6 B1 and C1). Correspondingly, b changes from decreasing to rising with increasing productivity (Fig.6 B2 and Fig.6 C2). However, the PSRR form and change in b are not very sensitive to the changes in g (Fig.6 B3 and B4; Fig.6 C3 and C4). With respect to the negative form, s_0=50 (i.e., high primary s) causes b to obviously increase for all four curves of PSRR (Fig.6 D2), leading to a rapid decline in s (Fig.6 D1). Different g values also lead to a great variation among the four negative PSRR forms (Fig.6 D3), but the change in b is relatively small (Fig.6 D4).

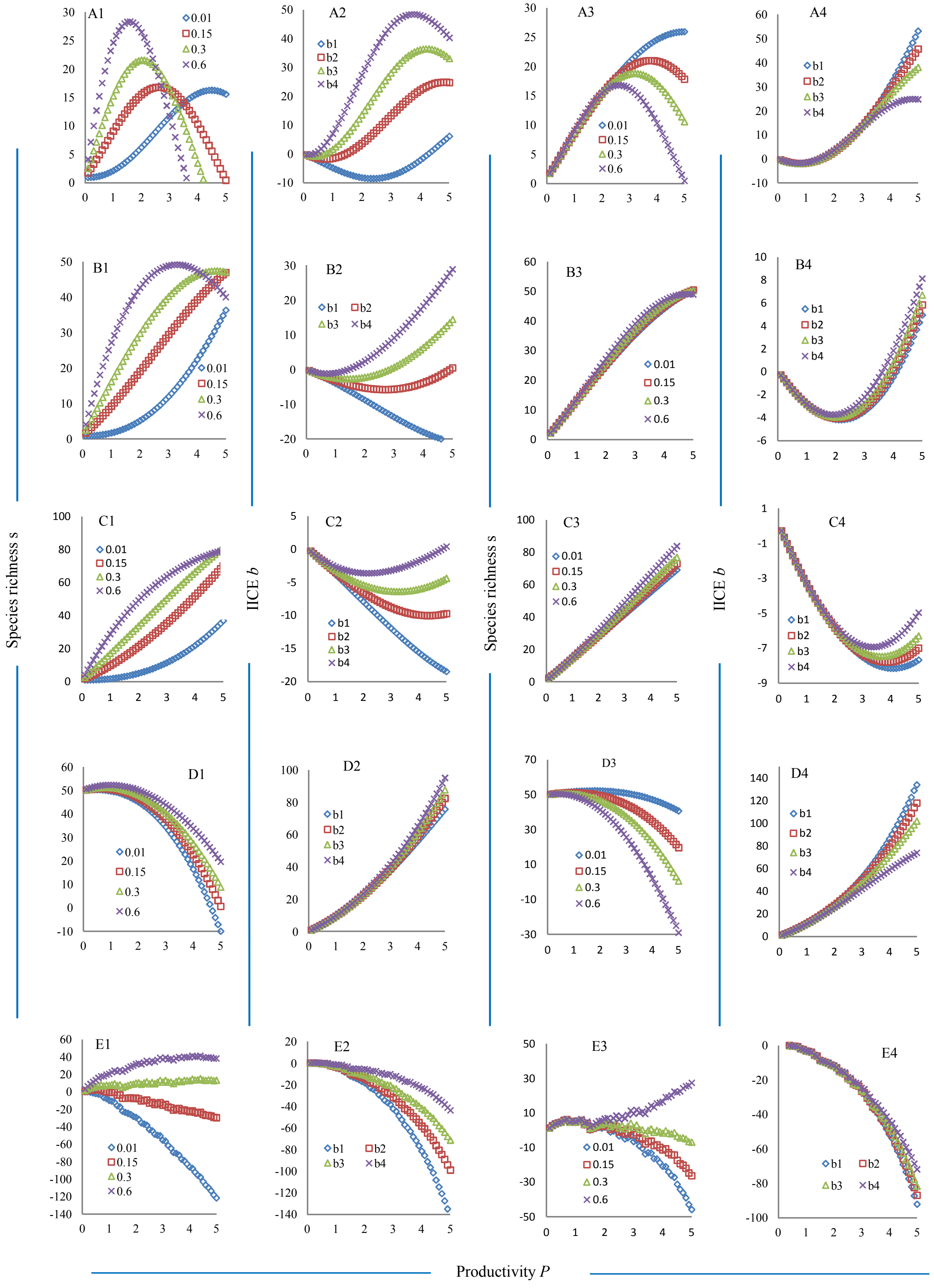

Figure 6 Responsive forms of PSRR under different strengths of two crucial processes: resource availability (m_2) and IICE (intra-and inter-specific competition effects) coefficient on

species richness (g) when ecosystem restoration temporally and spatially begins from a primary succession (Except D among the five forms). Modeling is performed by assigning the values of m_2 and g as 0.01, 0.15, 0.30, and 0.60 and s_i=0 for A, B, C and E but s_i=50 for D in equations 10 and 11. Other parameters are the same as the first value in each cell in the data columns marked with # in Supplemental Table 1. A1, B1, C1, D1, and E1 represent the responsive forms of PSRR to the humped, asymptotic, positive, negative, and irregular forms of PSRR in Figure1A1-E1 with changes in m_2, and A2, B2, C2, D2, and E2 are the dynamics of b, i.e., IICE, corresponding to these responsive forms (i.e., A1-E1). A3, B3, C3, D3, and E3 are the responsive forms of PSRR to the five typical forms of PSRR with changes in g, and A4, B4, C4, D4, and E4 are the dynamics of b corresponding to these responsive forms (i.e., A3–E3).

Notably, there are three responsive forms of PSRR with different m_2: negative, irregular, and asymptotic forms for the irregular form under the conditions of great variation in disturbance (Fig.6 E1, but no negative in the natural world).The shapes of PSRR also present three types—positive, irregular, and negative—with g being assigned different values (Fig.6 E3). The dynamics of b are different from each other for the different m_2 and g (Fig.6 E2 and E4). The reason for these complex changes in s and b is that, when there are disturbances with different intensities, the disturbance restricts both s and b. Consequently, weakened b and increasing m_2 promote an increase in s, which results in a complex feedback. Thus, the forms and dynamics of irregular PSRR are diverse, and the disturbance confounds PSRR.

The SRPR with variable m_2 and g also show different forms, but they all are not typically humped, asymptotic, positive, negative, and irregular forms (Fig.7 A1-E1 and Fig.7 A2-E2). The SRPR forms of the feedback relationships to the humped PSRR only show humped imprints (Fig.7 A1 and A2). The productivity peak of the curves occurs at a smaller s level with larger m_2 and g. The SRPR curve almost becomes leveled when m_2 or g equals 0.6 (Fig.7 A1 and A2). Comparatively, the SRPR forms to the asymptotic PSRR are closer to the humped forms except the curve where m_2 is assigned 0.01 (Fig.7 B1). The SRPR with different g includes positive, humped, and asymptotic forms (Fig.7 B2). The SRPR forms to the positive PSRR nearly present level lines with increasing s when m_2 and g are assigned different values (Fig.7 C1 and C2). The SRPR form to the negative PSRR where m_2 is respectively assigned 0.01 is relatively a typical negative form, but other curves are only various humped imprints (Fig.7 D1 and D2). These characteristics different from other SRPR forms are mainly due to high primary species richness (s_0=50). For the SRPR form to the

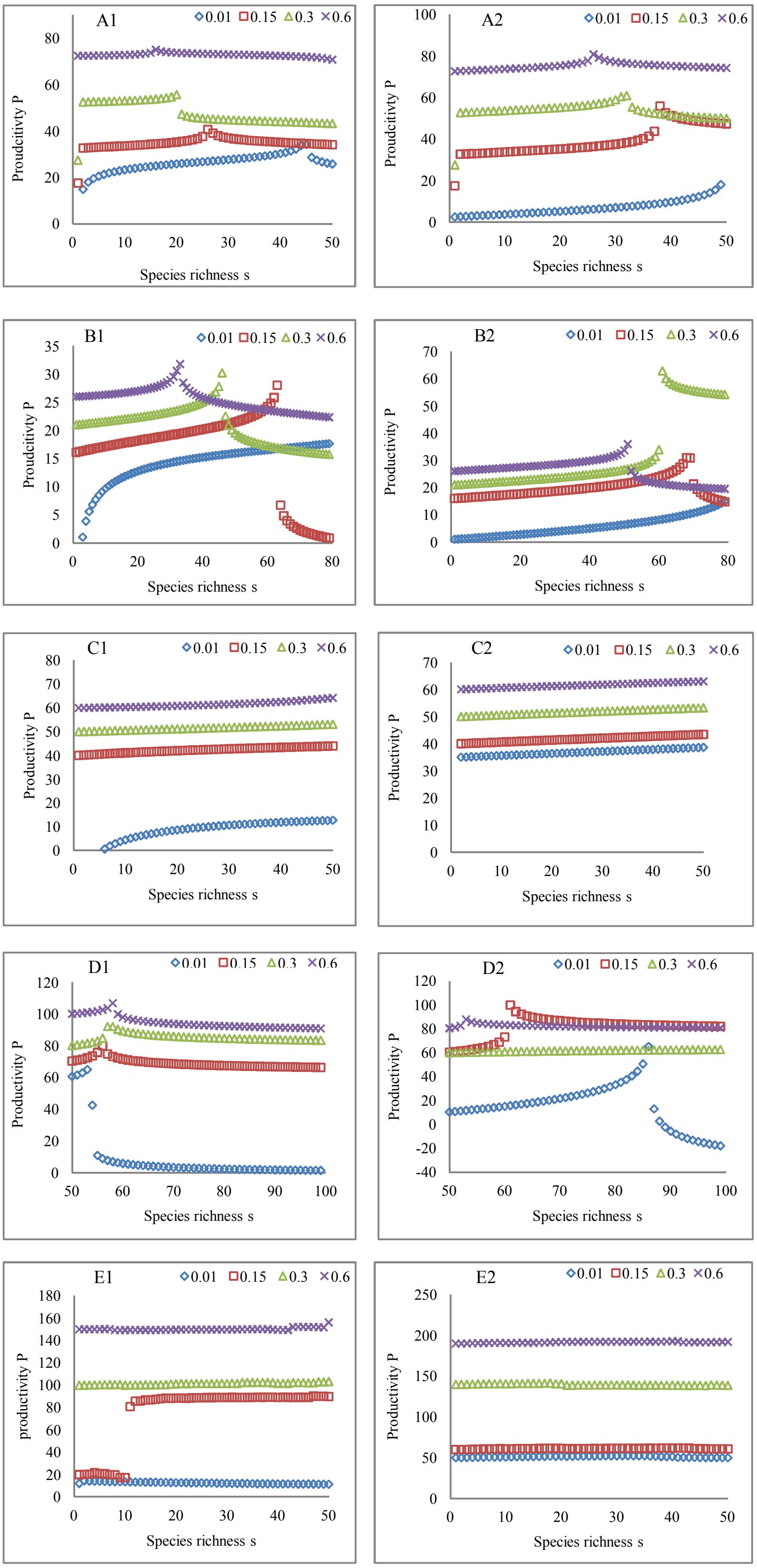

Figure 7 Responsive forms of SRPR under different strengths of two crucial processes: resource availability (m_2) and IICE coefficient on species richness (g) when ecosystem restoration temporally and spatially begins from a primary succession. In modeling, the assignment of all parameters is the same as that in Figure 6, but these parameters are substituted into equation 21. P_0 is considered to increase from a low to high level of m_2 and IICE (g), i.e., 0.01-0.60, in succession. A1, B1, C1, D1, and E1 represent the responsive forms of the humped, asymptotic, positive, negative, and irregular SRPR in Figure 2 A1-E1 to changes in m_2, and A2, B2, C2, D2, and E2 are the responsive forms of the five types of SRPR to changes in g. The dynamics of b to changes in m_2 and g are the same as those in Figure 6 A2-E2 and A4-E4.

irregular PSRR, the productivity maintains a level line with increasing *s* and disturbances, indicating that *s* does not play a substantial role in the regulation of SRPR (Fig.7 E1 and E2). These results indicate that the SRPR form is sensitive to the changes in m_2 or *g*, but is not especially sensitive to increasing *s*. Resource availability m_2, and *g* that reflects intra-and inter-specific competition effects, which have played a great role in productivity, may have to a great extent screened the effects of increasing *s* on SRPR here. Moreover, *g* is beneficial to maintain high productivity.
